# Supplementary material for: Characterization of Excited-State Electronic Structure in Diblock π-Conjugated Oligomers with Adjustable Linker Electronic Coupling
Source: Molecules. 2024 Jun 5;29(11):2678. doi: 10.3390/molecules29112678 (PMC11173629; doi:10.3390/molecules29112678)
Supplement: Supplementary file 1 [file molecules-29-02678-s001.zip › molecules-3003192-supplementary.pdf]

Supplementary Materials

Characterization of Excited-State Electronic Structure in Diblock π-Conjugated Oligomers with Adjustable Linker Electronic Coupling

Habtom B. Gobeze, Muhammed Younus, Michael D. Turlington,

Sohel Ahmed and Kirk S. Schanze*

Department of Chemistry, University of Texas at San Antonio

One UTSA Circle, San Antonio, TX 78249

Corresponding Author email: kirk.schanze@utsa.edu

Synthesis

General Methods. All reactions were carried out under a dry nitrogen atmosphere using dry solvents which were obtained from purification columns. Solvents for flash columns were obtained from commercial sources and used without any further purification. CuI was purchased from Sigma-Aldrich. TBT,[1] TBT-Br,[1] TBT-CC-TIPS,[1] TBT-CC-H,[1] T_4,[2] and T_4-$SnBu_3$,[3] *trans*-$[Pt(PBu_3)_2Cl(TBT)]$,[4] and TBBr[5] were prepared using literature methods.

Synthesis of ET5

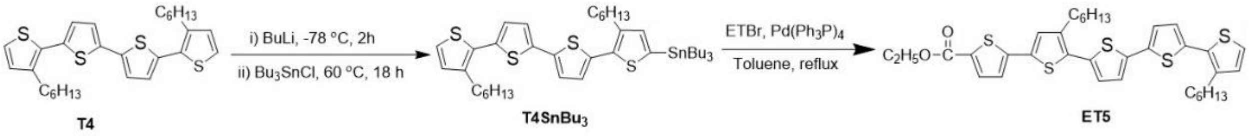

Scheme S1. Synthesis of ET5

Synthesis of ET5

To a solution of $\mathbf{T_4}$ (0.998 g, 2 mmol) in THF (10 mL) was added 2.5 M ^{n}BuLi (0.9 mL, 2.2 mmol) at -78 ºC and the solution was stirred for 1.5 h. Then Bu_3SnCl (0.716 g, 2.2 mmol) in THF (10 mL) was added under nitrogen atmosphere through a canula. The reaction was stirred under reflux for 18 h. After quenching the reaction with 1 mL water, 50 mL DCM was added, and the solution was washed with brine (50 mL x 3) and dried over $MgSO_4$. After removal of solvent at reduced pressure, the crude $\mathbf{T_4SnBu_3}$ was dried under vacuum (1.8 g) which was used without further purification. The crude T_4SnBu_3 was dissolved in toluene (10 mL), ethyl-5-bromothiophene-2-carboxylate and $Pd(PPh_3)_4$ (0.115 mg 5 mol%) were added and the reaction was stirred for 24 h under reflux. The solvent was evaporated, and the crude red oil was chromatographed on a flash column (silica) using 70/30 hexane/DCM. Yield: 0.586 g, 45% (overall in two steps). ^{1}H NMR (500 MHz, ($CDCl_3$): δ 7.71 (d, 1H), δ 7.20 (d, 1H), δ 7.17 (d, 1H), δ 7.16 (d, 1H), δ 7.15 (s, 1H), δ 7.14 (d, 1H), δ 7.08 (d, 1H), δ 7.05 (d, 1H), δ 6.96 (d, 1H), δ 4.38 (q, 2H), δ 2.79 (bm, 4H), δ 1.69 (m, 4H), δ 1.43 (m, 7H), δ 1.34 (m, 8H), δ 0.93 (m, 6H). ^{13}C NMR (125 MHz, $CDCl_3$): δ 14.14, 14.38, 22.62, 29.22, 29.31, 29.48, 30.43, 30.64, 31.65, 31.67, 61.23, 123.69, 123.93, 124.05, 126.56, 126.80, 128.09, 130.11, 130.23, 131.26, 131.70, 134.01, 134.13, 134.39, 135.65, 136.49, 137.36, 139.96, 140.73, 143.75, 162.08. Calc. for $C_{35}H_{40}O_2S_5$ (M/z): 652.1626, Found: 652.1624.

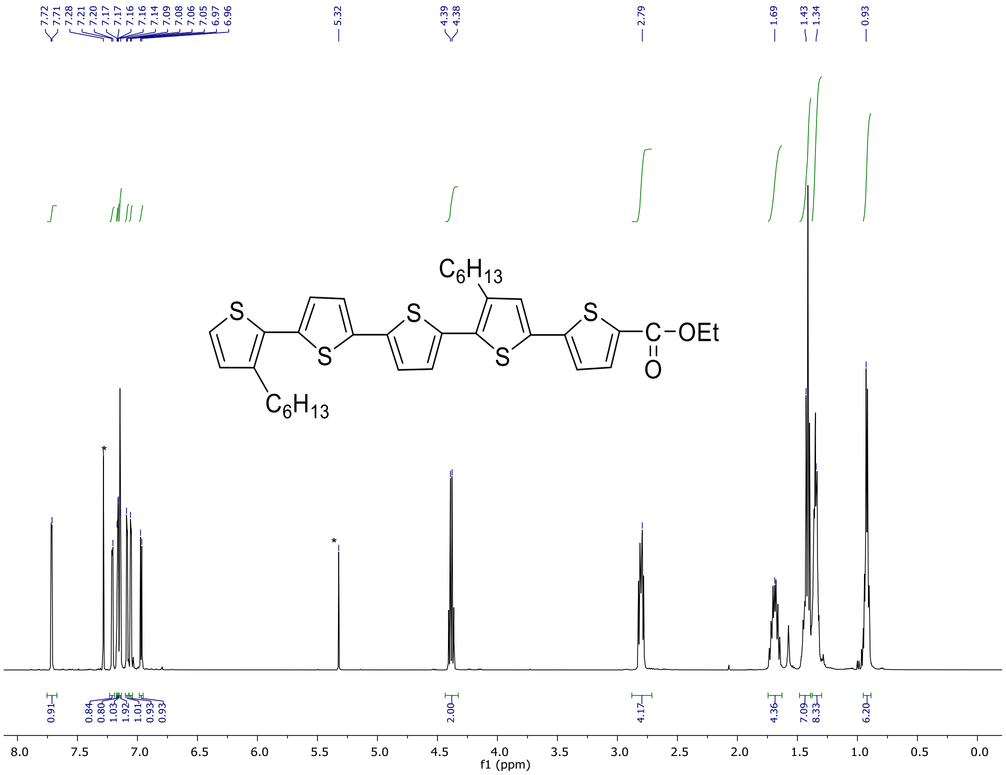

Figure S1. ^{1}H NMR of **ET5** in $CDCl_3$. *Solvent peak

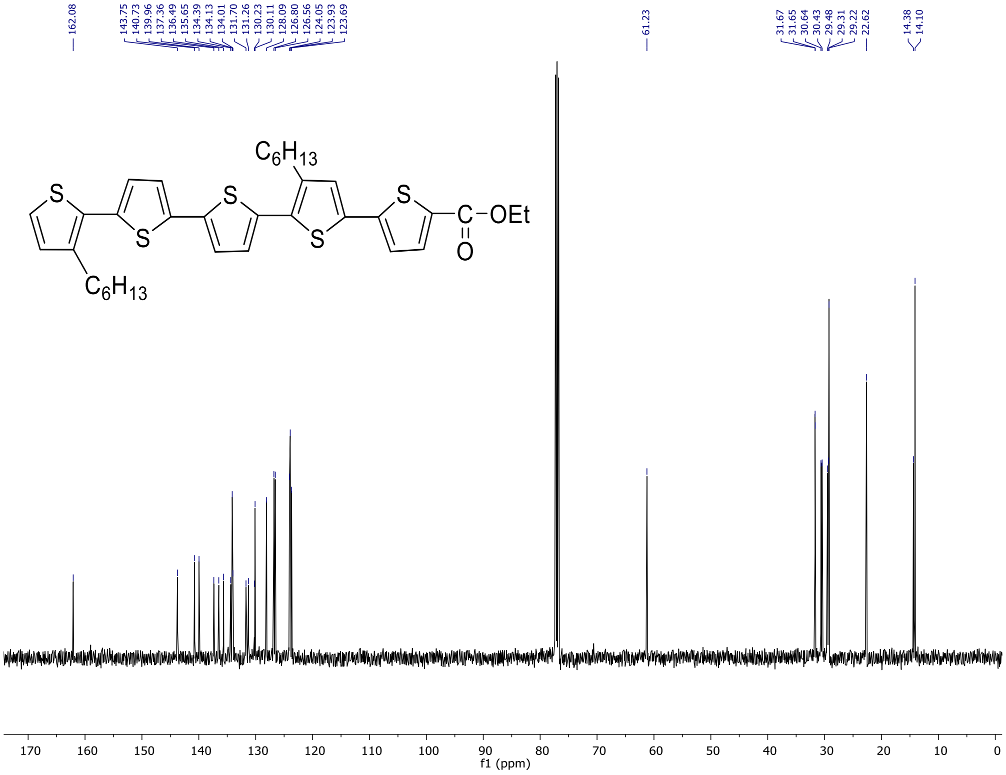

Figure S2. ^{13}C NMR of **ET5** in $CDCl_3$.

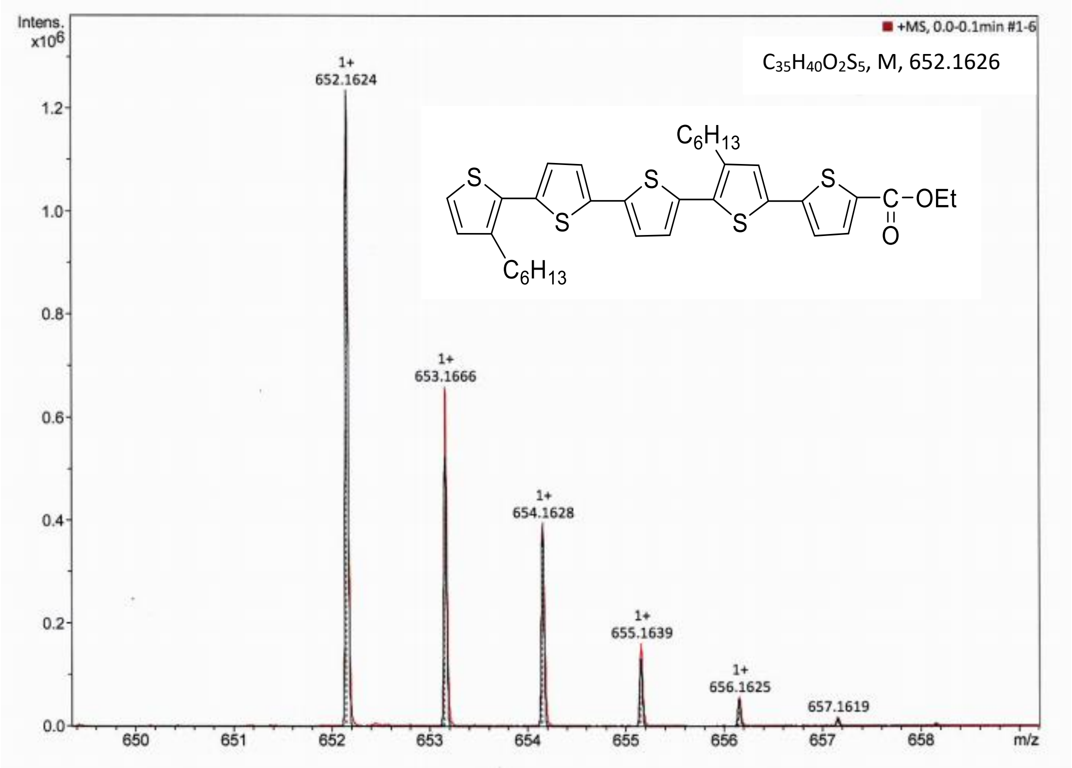

Figure S3. HRMS of **ET5**.

Synthesis of ET5TBT

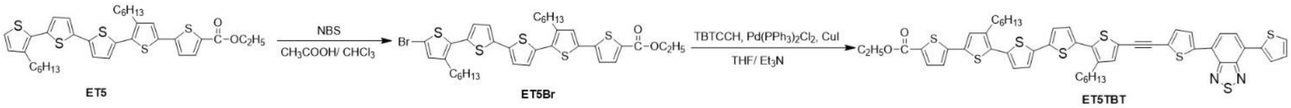

Scheme S2. Synthesis of **ET5TBT**

Synthesis of ET5Br

N-Bromosuccinimide (NBS, 0.145 g, 0.817 mmol) was added to a $CHCl_3/CH_3COOH$ (25/10 mL) solution of T5E (0.505 g, 0.773 mmol) at 0 ^oC and the solution was stirred at 0-5 ^oC for 4 h. Then the reaction mixture was poured into ice and neutralized with $NaCO_3$. After separation of the organic layer, the solution was dried using $MgSO_4$. After removal of solvent, the crude residue was chromatographed with hexane/DCM (60/40). Yield: 0.550 g, 97%. ^1H NMR (500 MHz, ($CDCl_3$): δ 7.71 (d, 1H), 7.14-1.16 (bm, 4H), 7.08 (d, 1H), 6.99 (d, 1H), 6.96 (s, 1H), 4.39 (q, 2H), 2.79 (t, 2H), 2.74 (t, 2H), 1.61-1.73 (m, 4), 1.39-1.46 (m, 7H), 1.31-1.38 (m, 8H), 0.89-0.95 (m, 6H). ^{13}C NMR (125 MHz, $CDCl_3$): δ 162.7, 143.70, 140.81, 140.55, 137.07, 137.02, 134.67, 134.12, 134.10, 132.72, 131.75, 131.71, 131.14, 128.09, 127.00, 126.81, 124.14, 124.02, 123.72, 110.68, 61.24, 31.64, 31.62, 30.51, 30.43, 29.72, 29.48, 29.24, 29.21, 29.10, 22.60, 14.38, 14.10. Calc. for $C_{33}H_{35}BrO_2S_5$ (M/z): 730.0731, Found: 730.0727.

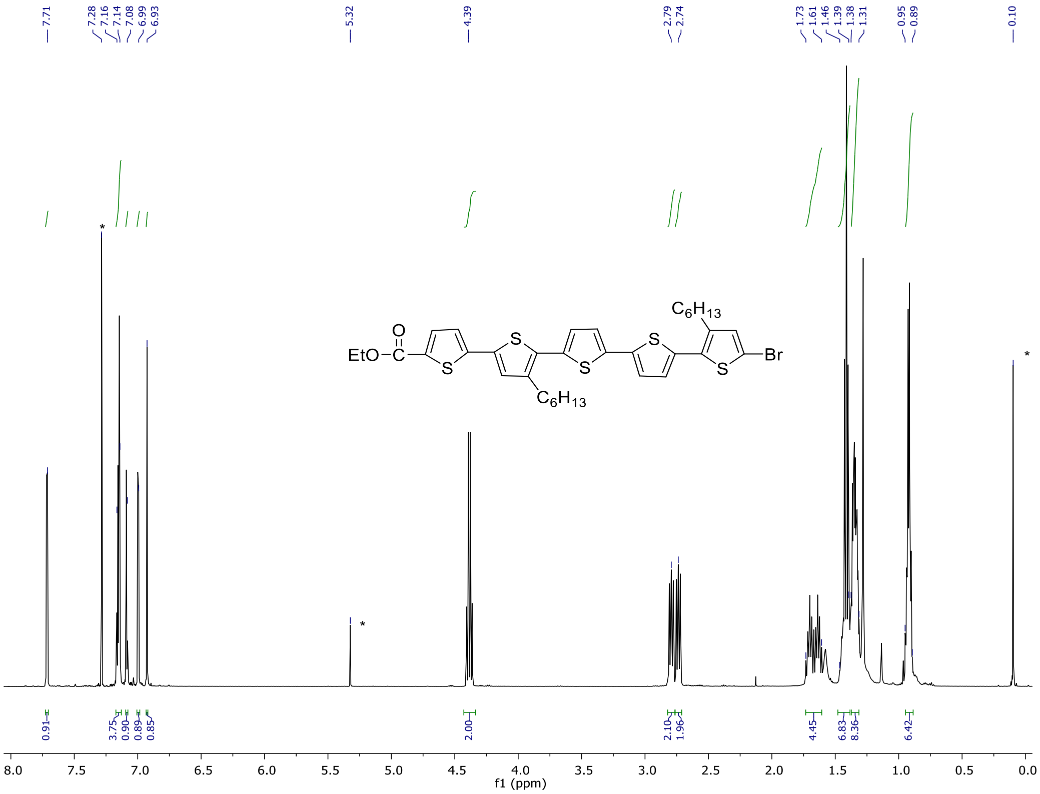

Figure S4. ^{1}H NMR of **ET5Br** in $CDCl_3$. *Solvent/impurity peak

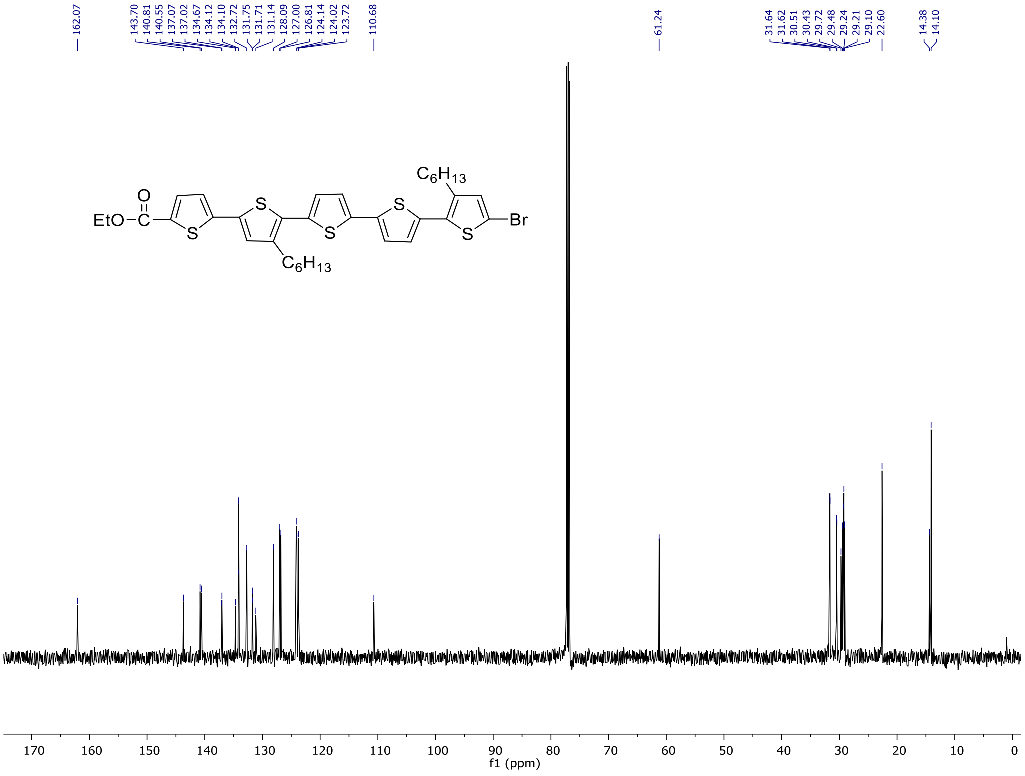

Figure S5. ^{13}C NMR of **ET5Br** in $CDCl_3$.

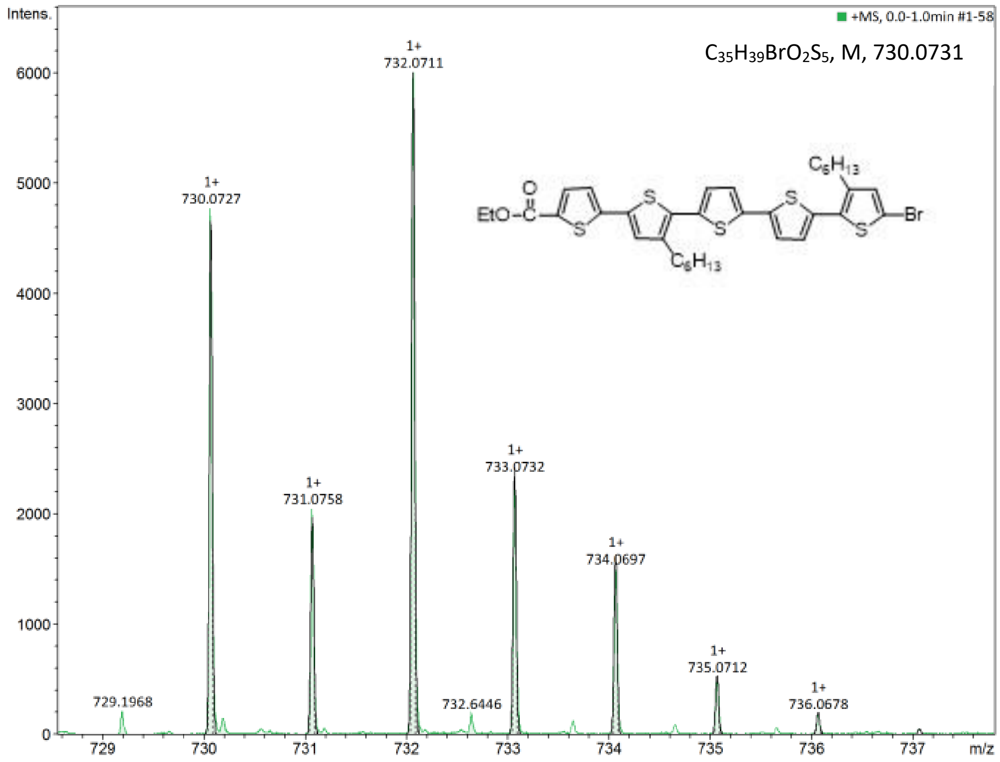

Figure S6. HRMS of **ET5Br**.

Synthesis of ET5TBT

To a solution of **ET5Br** (50 mg, 0.068 mmol) in THF/$^{iso}Pr_2NH_2$ (10/3 mL) were added TBTCCH (24 mg, 0.075 mmol), $Pd(PPh_3)_2Cl_2$ (2.5 mg, 5 mol%), CuI (1 mg, 5 mol%) and the reaction was stirred for 18 h at 75 ºC. After removal of solvents under reduced pressure, the residue was chromatographed on a flash column using hexane/DCM (3/2). Yield: 35 mg, 53%. ^{1}H NMR (500 MHz, ($CDCl_3$): δ 8.15 (d, 1H), 8.04 (d, 1H), 7.88 (b, 2H), 7.71 (d, 1H), 7.49 ((d, 1H), 7.36 (d, 1H), 7.24 (d, 1H), 7.16 (b, 3H), 7.14 (d, 2H), 7.09 (d, 2H), 4.32 (q, 2H), 2.79 (t, 4H), 1.70 (b, 4H), 1.41 (b, 7H), 1.35 (b, 8H), 0.92 (t, 6H). ^{13}C NMR (125 MHz, $CDCl_3$): δ 162.08, 152.57, 152.46, 143.70, 140.99, 140.79, 139.84, 139.25, 137.12, 137.02, 135.24, 134.71, 134.55, 134.13, 134.06, 132.87, 132.84, 131.72, 131.17, 128.10, 127.75, 127.30, 127.09, 126.98, 126.80, 126.48, 125.75, 125.65, 125.12, 124.18, 124.13, 123.71, 120.56, 88.39, 87.74, 61.25, 31.68, 31.65, 30.42, 30.37, 29.50, 29.36, 29.23, 29.18, 22.63, 14.38, 14.12. Calc. for $C_{51}H_{46}N_2O_2S_8$ (M/z): 974.1319, Found: 974.1302.

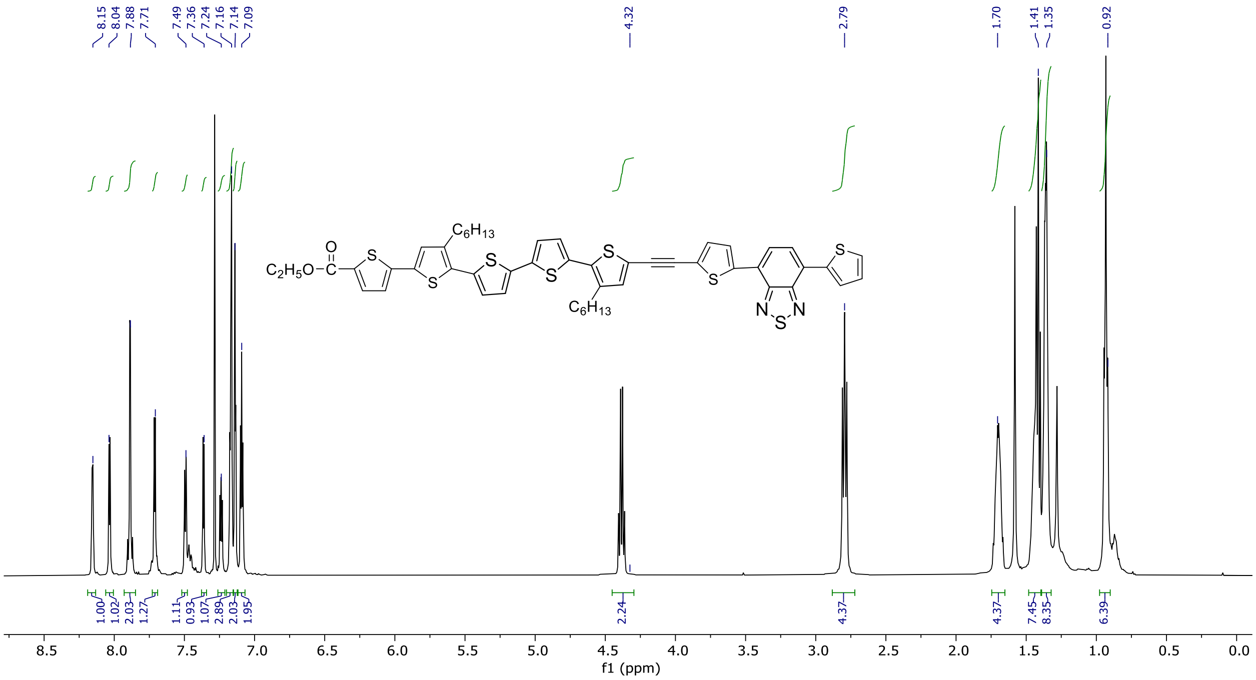

Figure S7. ^{1}H NMR of **ET5TBT** in $CDCl_3$.

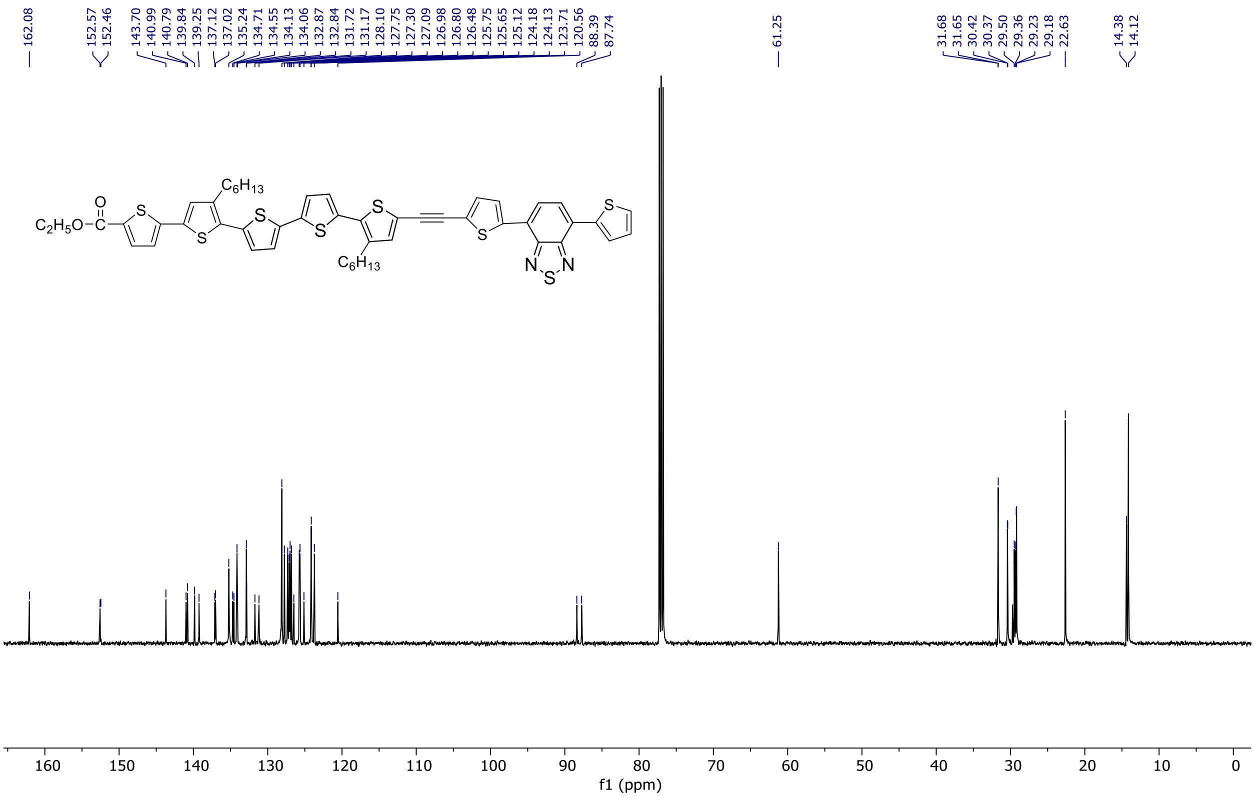

Figure S8. ^{13}C NMR of **ET5TBT** in $CDCl_3$.

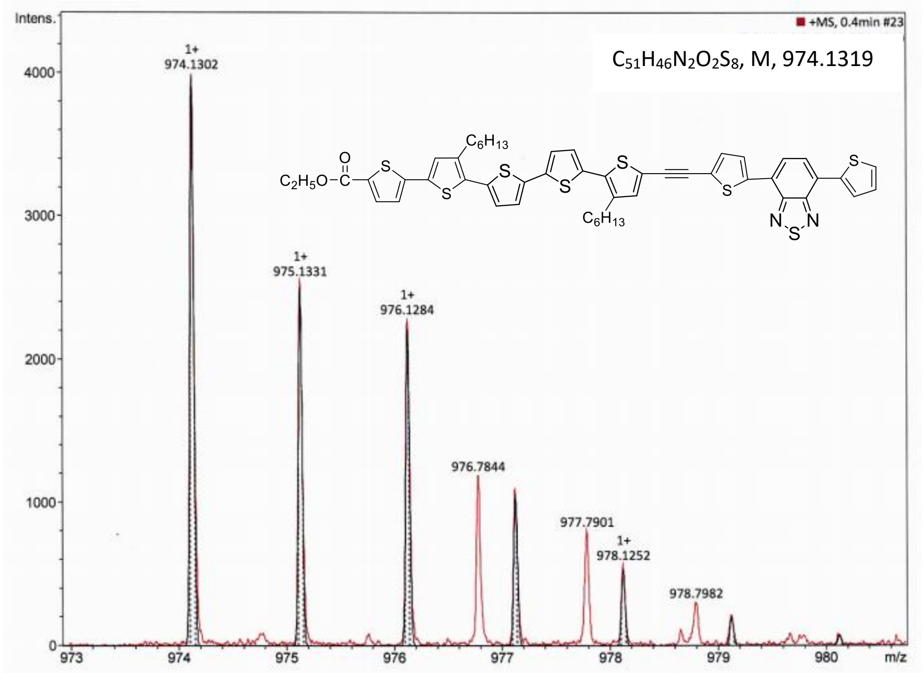

Figure S9. HRMS of **ET5TBT**.

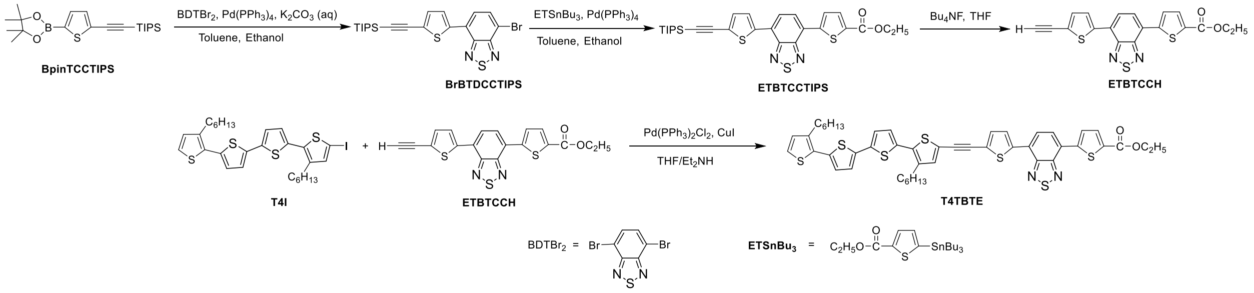

Scheme S3. Synthesis of **T4TBTE**

Synthesis of T4TBTE

Synthesis of BrBTDCCTIPS

BpinTCCTIPS (800 mg, 2.05 mmol), Br_2BTD (1.50 g, 5.13 mmol), and potassium bicarbonate (2M in H_2O, 10 mL) were added to 40 mL of THF. Four cycles of freeze-pump-thaw were performed to degas the solution before adding $Pd(PPh_3)_4$ (230 mg, 200 μmol) under N_2 protection. The solution was refluxed for 24 hours. The reaction was quenched by adding water. The mixture was rinsed with deionized water and brine and extracted several times with DCM. The organic layer was dried over Na_2SO_4. An orange solid (450 mg, 47%) was obtained after purification by column chromatography with hexanes/DCM (6/1). ^{1}H NMR (500 MHz, ($CDCl_3$): δ 7.91 (d, 1H), 7.82 (d, 1H), 7.66 (d, 1H), 7.29 (d, 1H), 1.10 (b, 21 H) ppm. ^{13}C NMR (125 MHz, $CDCl_3$): δ 153.71, 151.58, 139.22, 133.18, 132.16, 127.44, 126.33, 125.69, 125.50, 112.89, 99.20, 99.08, 18.69, 11.33 ppm.

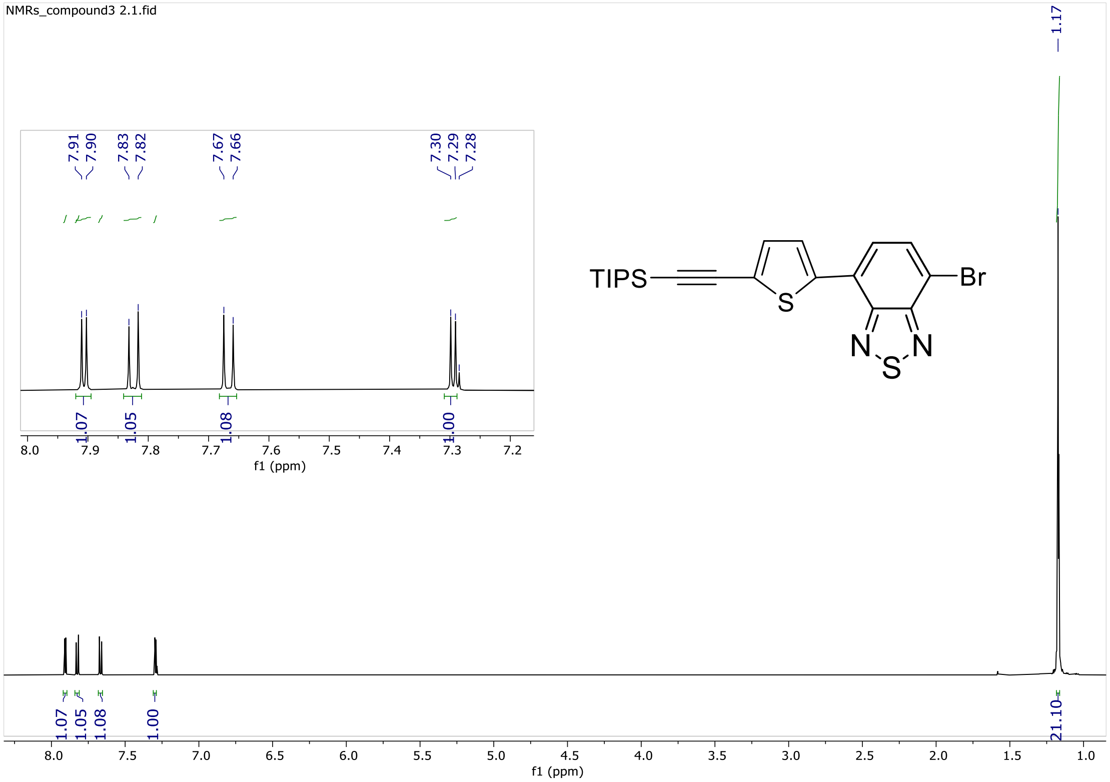

Figure S10. ^{1}H NMR of **BrBTDCCTIPS** in $CDCl_3$.

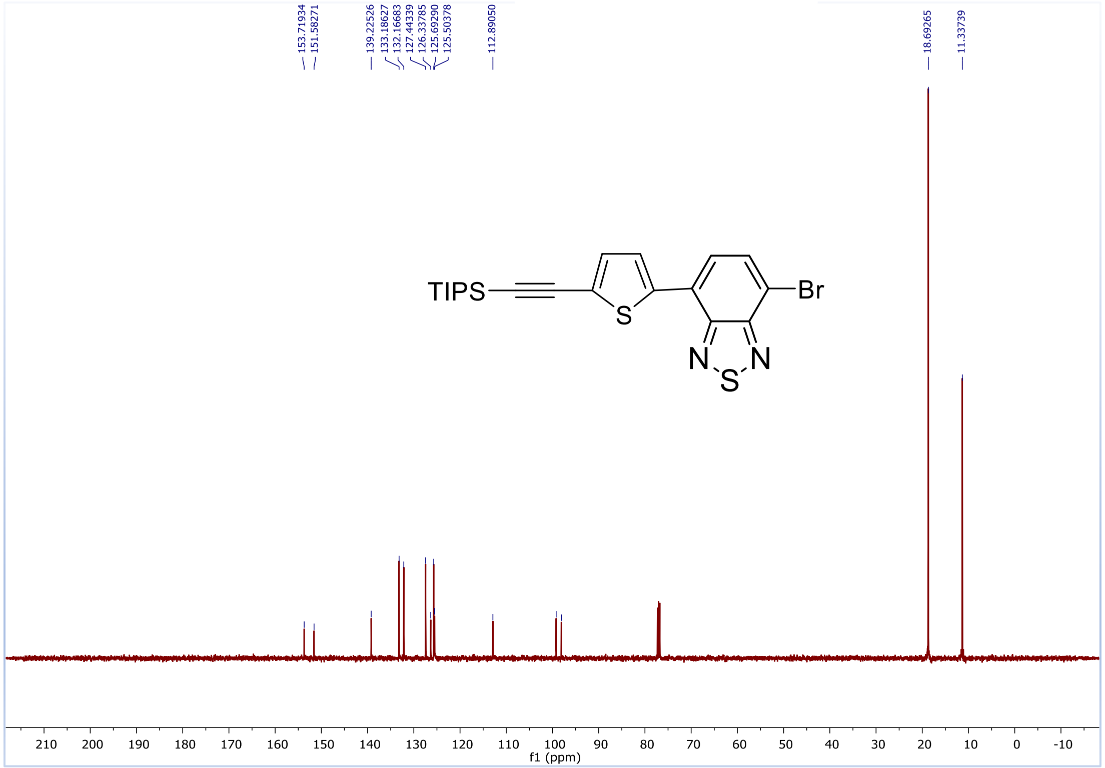

Figure S11. ^{13}C NMR of **BrBTDCCTIPS** in $CDCl_3$.

Synthesis of ETBTCCTIPS

Compound **ETSnBu3** (300 mg, 629 μmol) and **BrBTDCCTIPS** (429 mg, 942 μmol) were added to a Schlenk flask containing 15 mL THF. Four cycles of freeze-pump-thaw were performed to degas the solution before adding $Pd(PPh_3)_4$ (72 mg, 63 μmol) under N_2 atmosphere. The reaction was refluxed for 24 hours and quenched with 2 mL water. Then the mixture was washed with deionized water and brine and extracted several times with DCM. The organic layer was dried over Na_2SO_4. A red solid (260 mg, 75%) was obtained after purification by column chromatography with hexanes/DCM (6/1). ^1H NMR (500 MHz, ($CDCl_3$): 7.99 (d, 1H), 7.93 (d,1H), 7.86 (d, 1H), 7.84 (d, 1H), 7.79 (d, 1H), 7.30 (d, 1H), 4.44 (q, 2H), 1.47 (t, 3H), 1.21 (b, 21 H). ^{13}C NMR (125 MHz, $CDCl_3$): δ 162.23, 152.28, 152.25, 145.27, 139.77, 134.29, 133.75, 133.21, 127.40, 126.44, 126.40, 125.55, 125.24, 125.13, 99.36, 98.15, 61.31, 18.70, 14.42, 11.35.

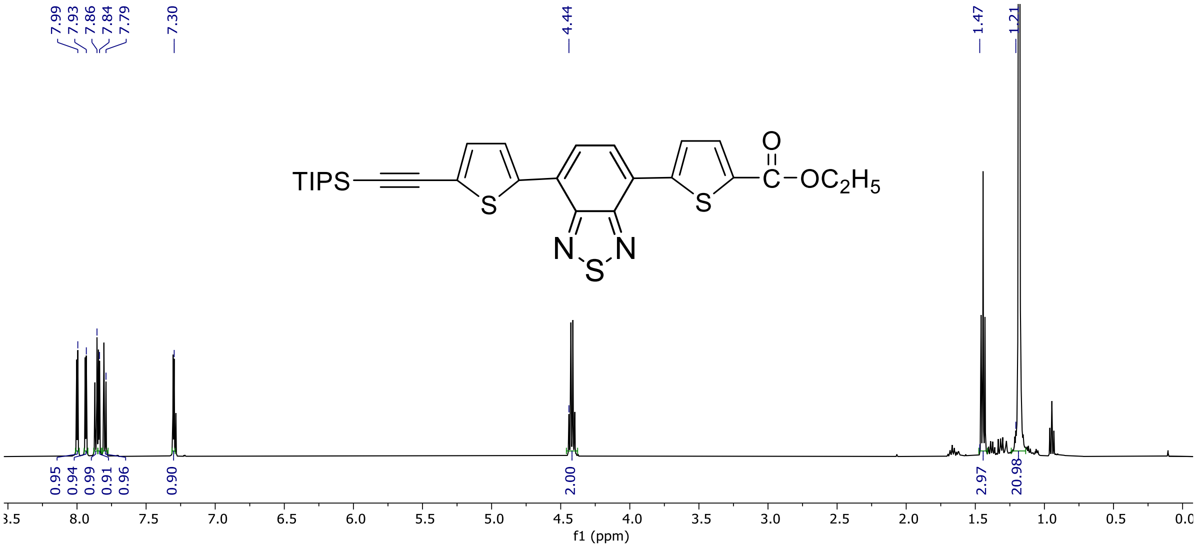

Figure S12. ^1H NMR of **ETBTCCTIPS** in $CDCl_3$.

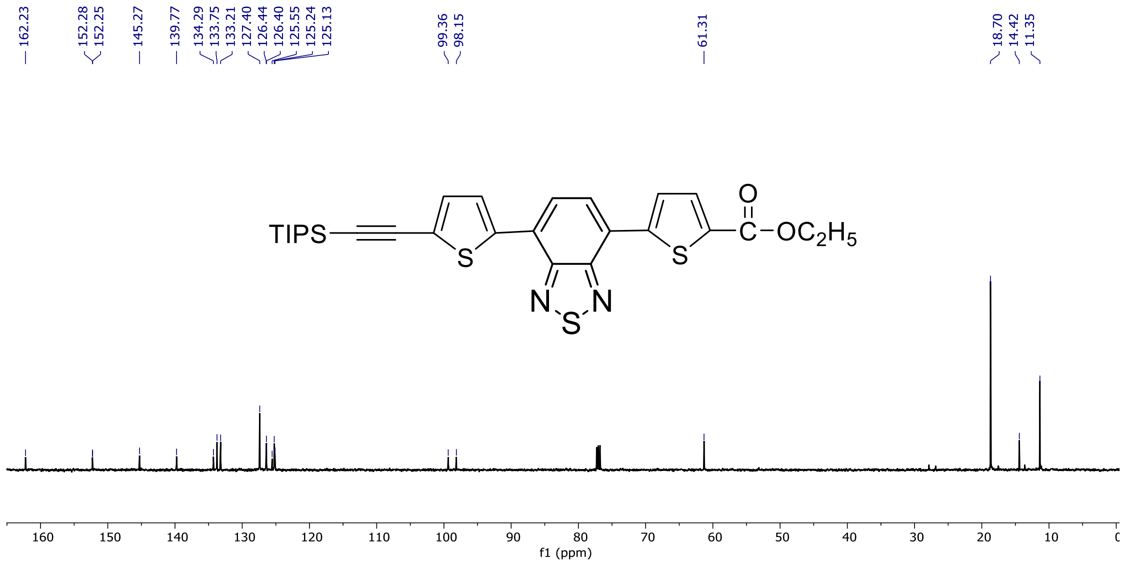

Figure S13. ^{13}C NMR of **ETBTCCTIPS** in $CDCl_3$.

Synthesis of ETBTCCH

To a solution of ETBTCCTIPS (100 mg, 0.18 mmol) in THF (10 mL) was added Bu_4NF at 0 °C. After raising the temperature to RT, the reaction was stirred for 30 minutes and then quenched with water. The solvent was then removed under reduced pressure, and the residue was redissolved with DCM, dried over $MgSO_4$, and DCM was removed. The residue was chromatographed on flash column using DCM/hexane to give a yellow solid. Yield: 49 mg, 71%. ^{1}H NMR (500 MHz, $CDCl_3$): 8.06 (d, 1H), 8.00 (d, 1H), 7.95 (d, 1H), 7.88-7.89 (m, 2H), 4.42 (q, 2H), 3.52 (s, 1H), 1.46 (t, 3H). ^{13}C NMR (125 MHz, $CDCl_3$): δ 162.29, 152.46, 152.37, 145.29, 140.47, 134.49, 133.95, 133.84, 127.55, 127.43, 126.38, 126.32, 125.59, 125.46, 123.76.

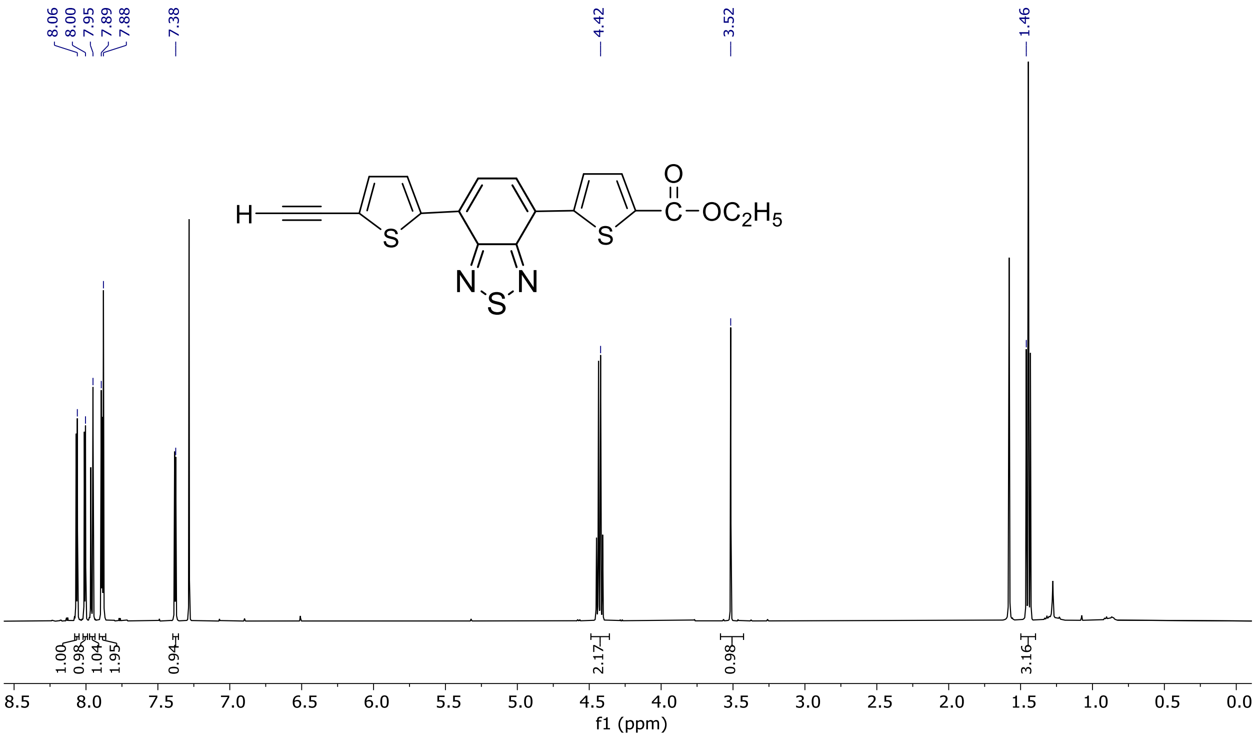

Figure S14. ^{1}H NMR of **ETBTCCH** in $CDCl_3$.

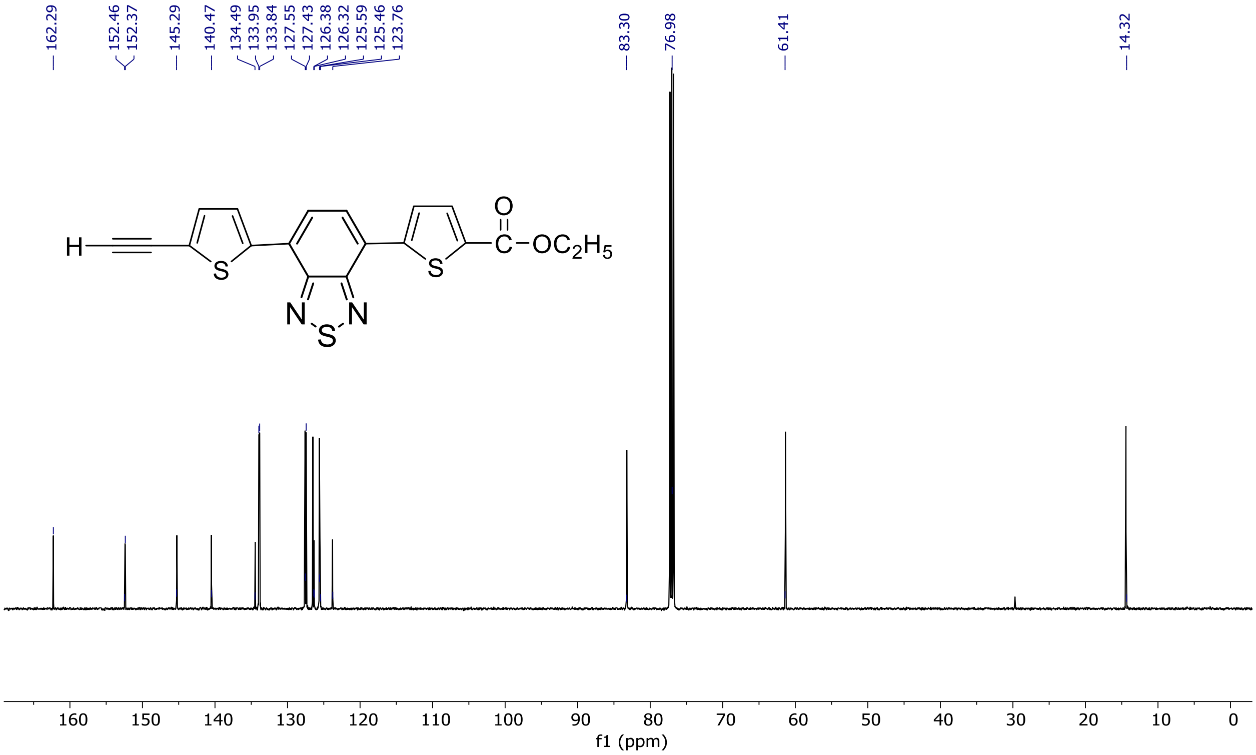

Figure S15. ^{13}C NMR of **ETBTCCH** in $CDCl_3$.

Synthesis of T4TBTE

To a solution of ETBTCCH (22 mg, 0.06) in THF/$^{iso}Pr_2NH$ (5/2 mL), were added T4I (30 mg, 0.05), $Pd(PPh_3)_2Cl_2$ (3.5 mg, 10 mol%) and CuI (1 mg, 10 mol%) under nitrogen atmosphere, and the reaction was stirred for 18 h at RT in which time the reaction was turned to red. The red solution was washed with brine water (10 mL x 2), dried over $MgSO_4$, and the solvents were removed under reduced pressure. The residue was chromatographed on flash column using 1/1 DCM/hexane, and the red band was collected. Yield: 22 mg, 51%. ^1H NMR (500 MHz, $CDCl_3$): 8.07 (d, 2H), 8.97 (d, 2H), 7.91 (s, 1H), 7.89 (b, 1H), 7.37 (d, 1H), 7.22 (d, 1H), 7.17 (b, 2H), 7.10 (d, 1H), 7.06 (d, 1H), 7.97 (d, 1H), 4.42 (q, 2H), 2.79 (b, 4H), 1.69 (b, 4H), 1.34-1.45 (bm, 15 H), 0.92 (b, 6H). ^{13}C NMR (125 MHz, $CDCl_3$): δ 162.30, 152.44, 152.36, 145.36, 140.55, 139.97, 139.80, 137.50, 136.45, 135.68, 135.33, 134.37, 134.18, 133.85, 133.09, 132.90, 130.22, 130.12, 127.51, 127.01, 126.57, 125.41, 125.34, 124.85, 124.11, 123.95, 123.92, 120.33, 88.77, 87.54, 61.35, 31.94, 31.67, 30.65, 30.39, 29.72, 29.51, 29.33, 29.31, 29.23, 29.17, 22.63, 14.42, 14.12. Calculated for $C_{47}H_{44}N_2O_2S_7$ (M/z): 892.1442; Found: 892.1442.

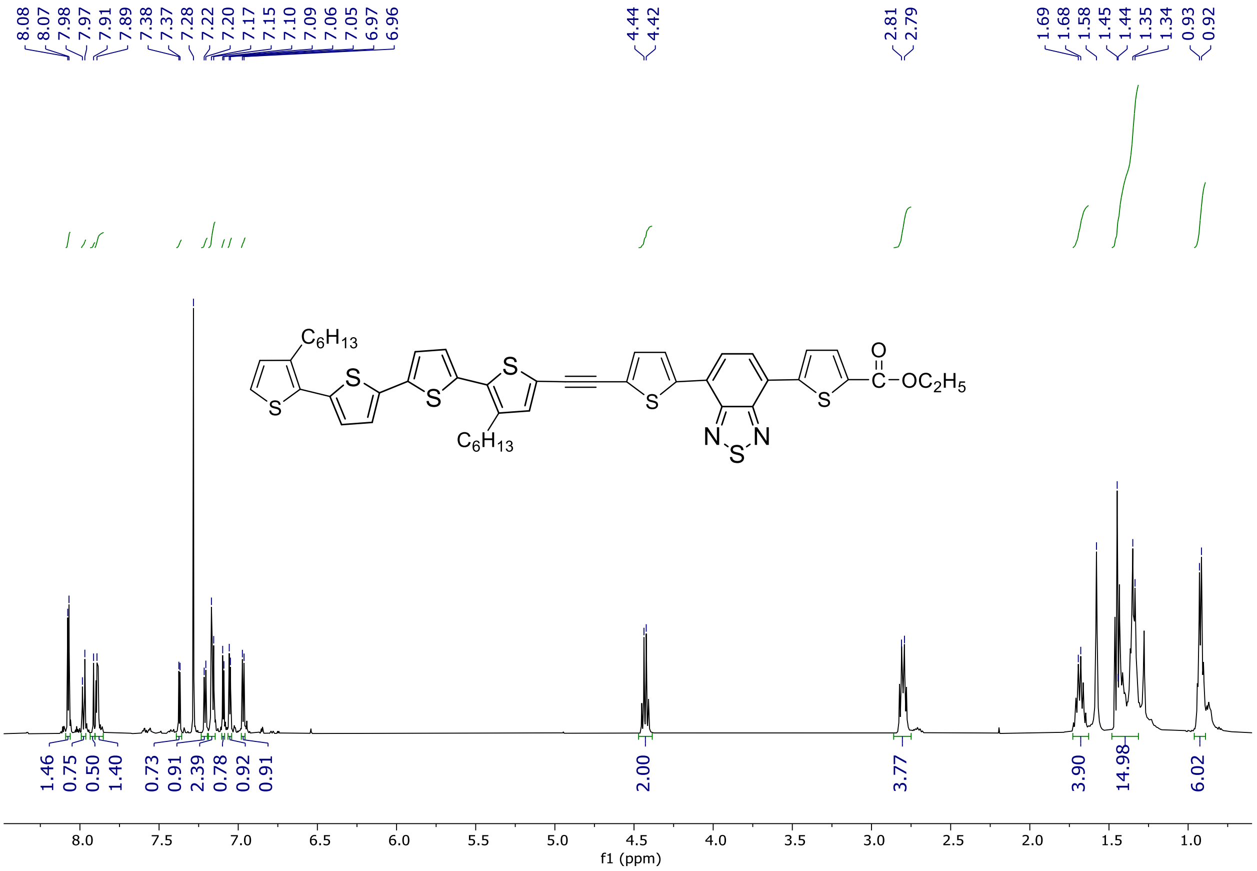

Figure S16. ^{1}H NMR of **T4TBTE** in $CDCl_3$.

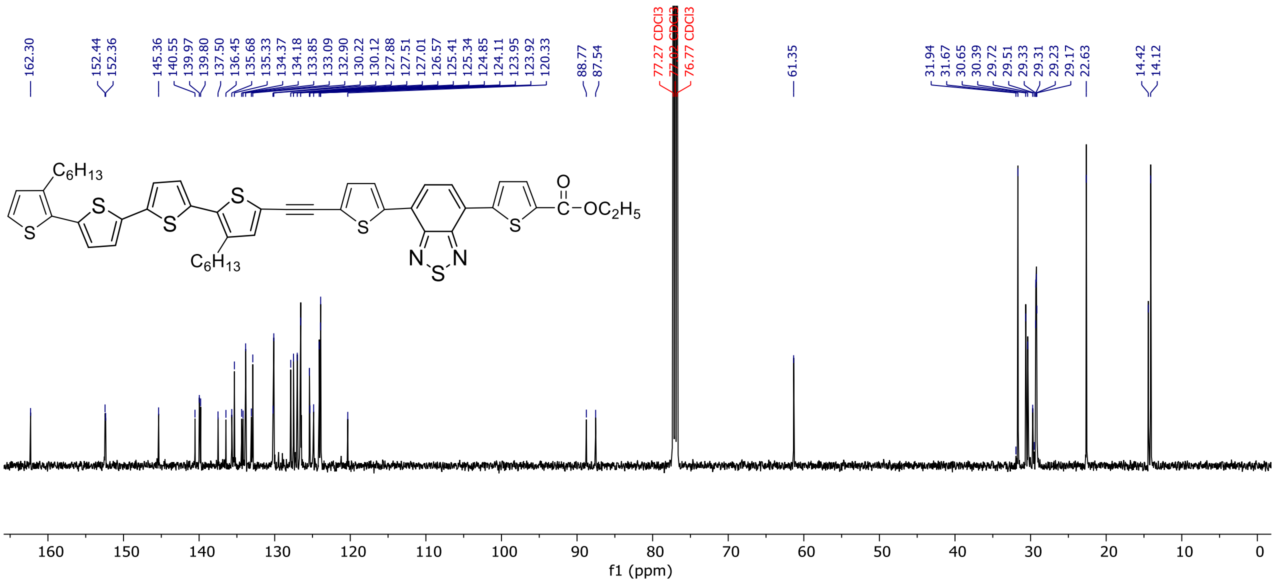

Figure S17. ^{13}C NMR of **T4TBTE** in $CDCl_3$.

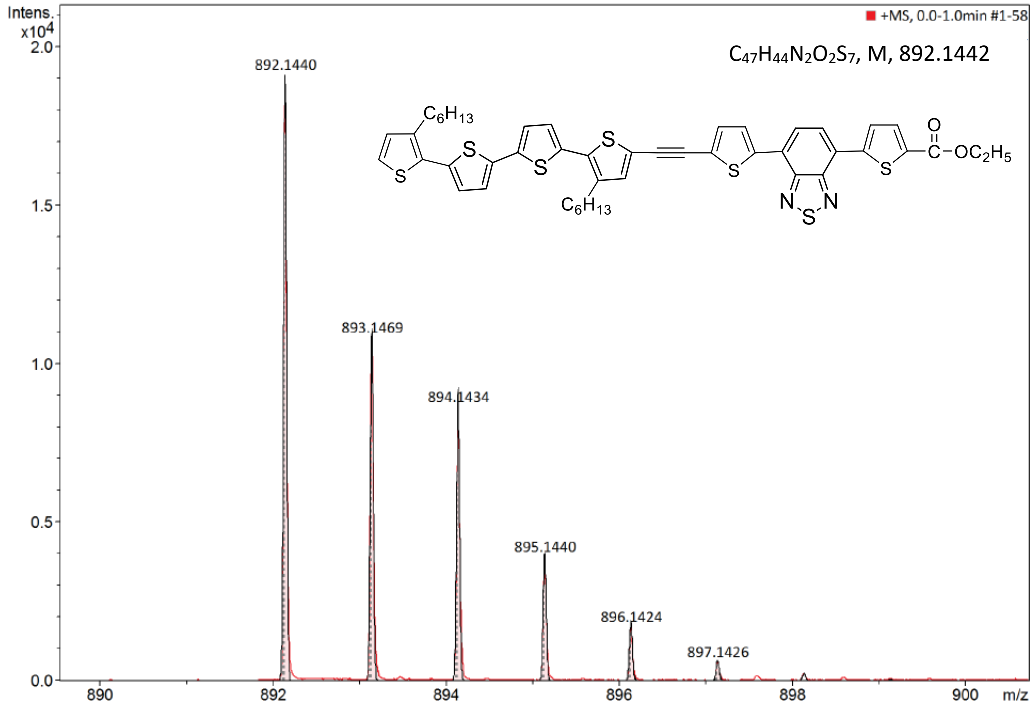

Figure S18. **HRMS of** ^{1}H NMR of **T4TBTE**.

1.1.4. Synthesis of TBTE

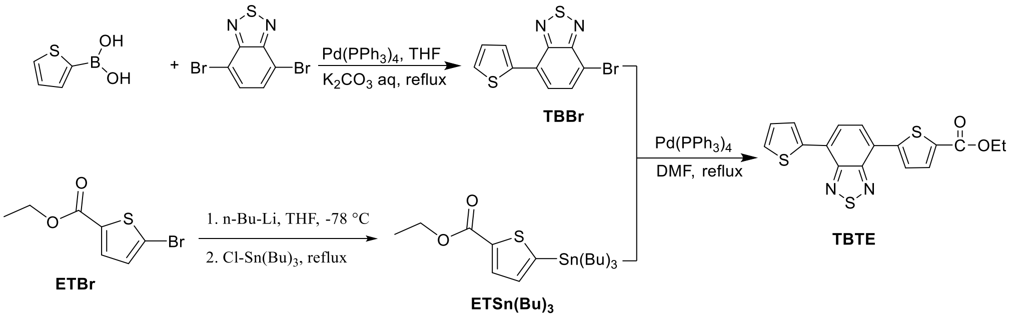

Scheme S4. Synthesis of **TBTE**

Synthesis of ETSn(Bu)$_3$

To a degassed solution of **ETBr** (500 mg, 2.12 mmol) in THF (5mL) was added 2.5 M n-BuLi (1.02 mL, 2.55 mmol) at -78 °C and the solution was stirred for 2 h. After that, Bu_3SnCl (830 mg, 2.55 mmol) in THF (2 mL) solution was added and the reaction mixture was refluxed overnight. The reaction was quenched by adding 1 mL of water and extracted with diethyl ether. The extracted layer was washed with brine and dried over anhydrous $MgSO_4$. The solvent was removed under reduced pressure. The crude product was purified with silica gel column chromatography (DCM/n-hexane 1:1) to obtain **ETSn(Bu)$_3$** (175 mg, Yield 35%) as a colorless oil. ^{1}H NMR (500 MHz, ($CDCl_3$): δ 7.89 (d, 1H), δ 7.16 (d, 1H), δ 4.36 (q, 2H), δ 0.8-1.3 (m, 30H).

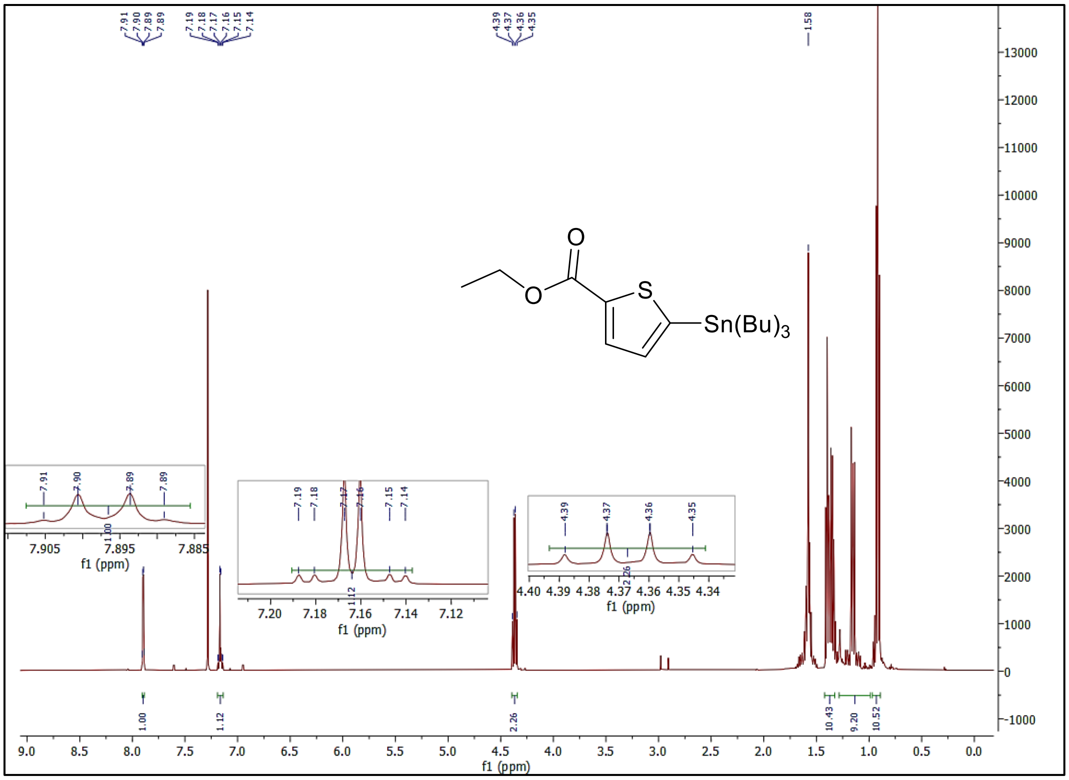

Figure S19. ^{1}H NMR of **ETSn(Bu)$_3$** in $CDCl_3$.

Synthesis of TBTE

Under inert conditions, **ETSn(Bu)$_3$** (170 mg, 0.38 mmol), **TBBr** (120 mg, 0.41 mmol) and dry DMF (7 mL) were added to a round bottom flask. After the addition of catalytic amount of $Pd(PPh_3)_4$ (5 mol %), the reaction mixture was refluxed for 16 h and then the solvent was removed under reduced pressure. The crude residue was purified with silica gel column chromatography with DCM/n-Hexane (1:1) eluent to obtain **TBTE** (72.5 mg, Yield 49%) as an orange amorphous solid. ^{1}H NMR (500 MHz, ($CDCl_3$): δ 8.18 (d, 1H), δ 8.06 (d, 1H), δ 7.97 (d, 1H), δ 7.92 (d, 1H), δ 7.89 (d, 1H), δ 7.51 (d, 1H), δ 7.25 (t, 1H), δ 4.42 (q, 2H), δ 1.45 (t, 3H). ^{13}C NMR (125 MHz, $CDCl_3$): δ 14.42, 61.32, 124.94, 125.49, 126.72, 127.35, 127.40, 127.44, 128.09, 128.16, 133.85, 134.16, 139.07, 145.55, 152.53, 152.56, 162.35.

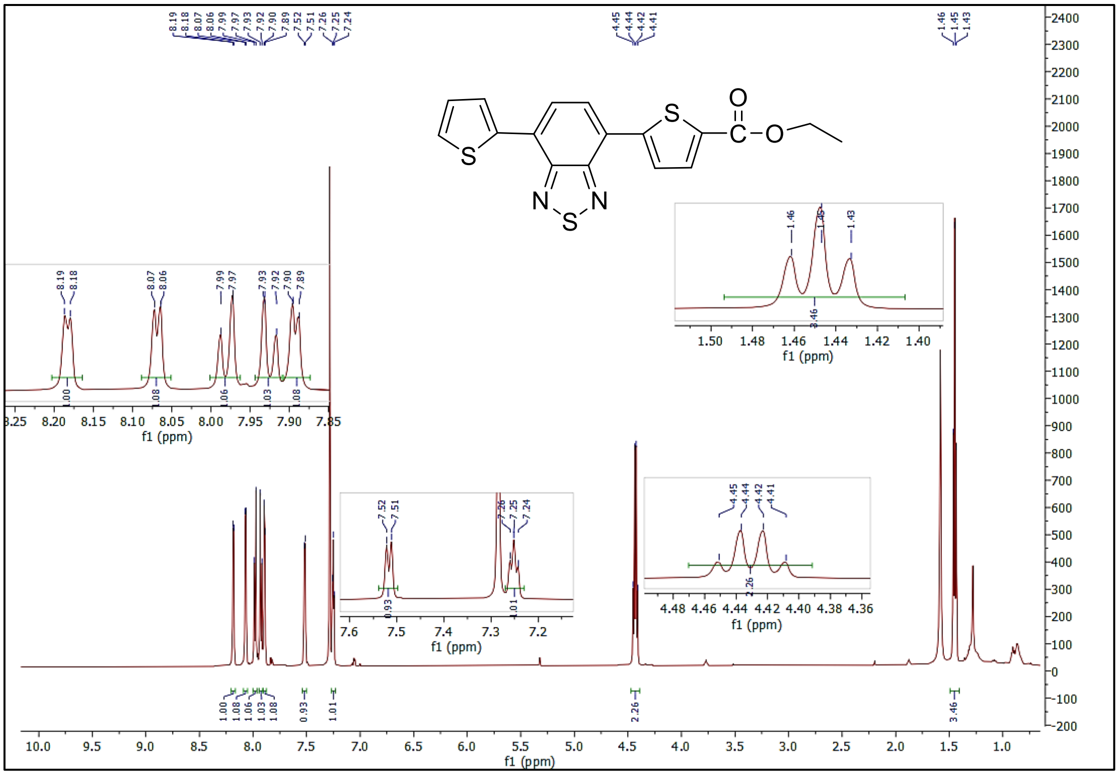

Figure S20. ^{1}H NMR of **TBTE** in $CDCl_3$.

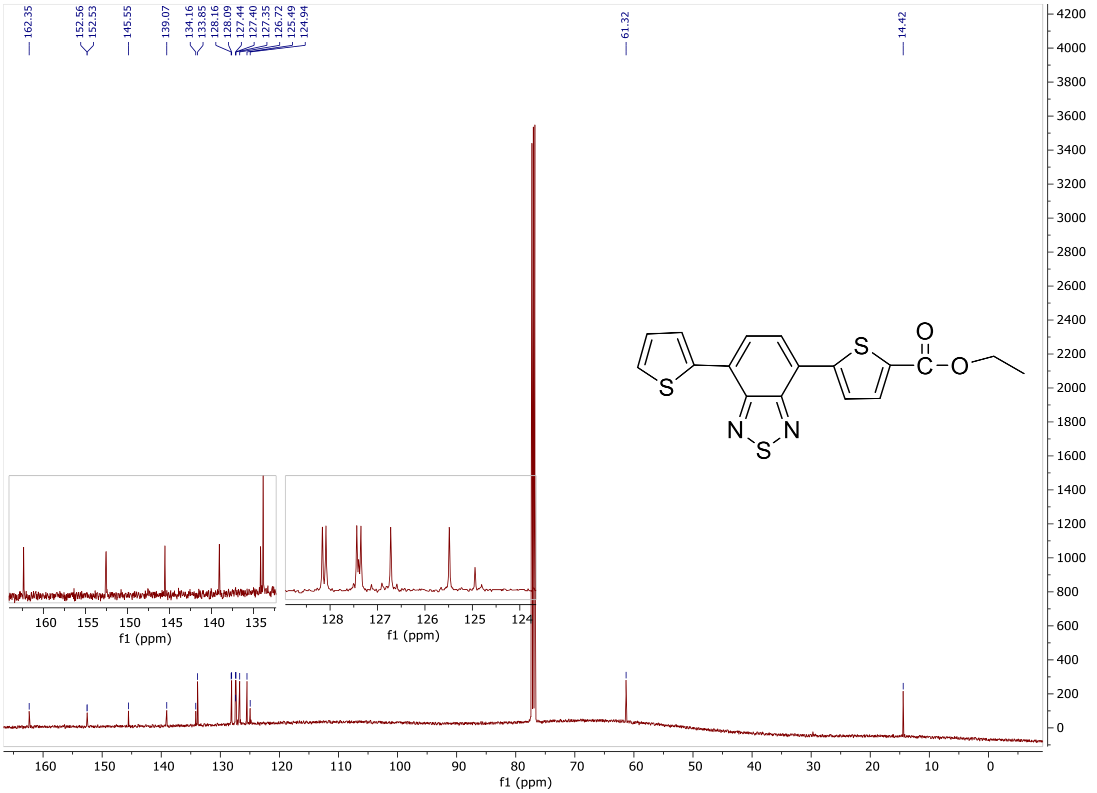

Figure S21. ^{13}C NMR of **TBTE in** $CDCl_3$.

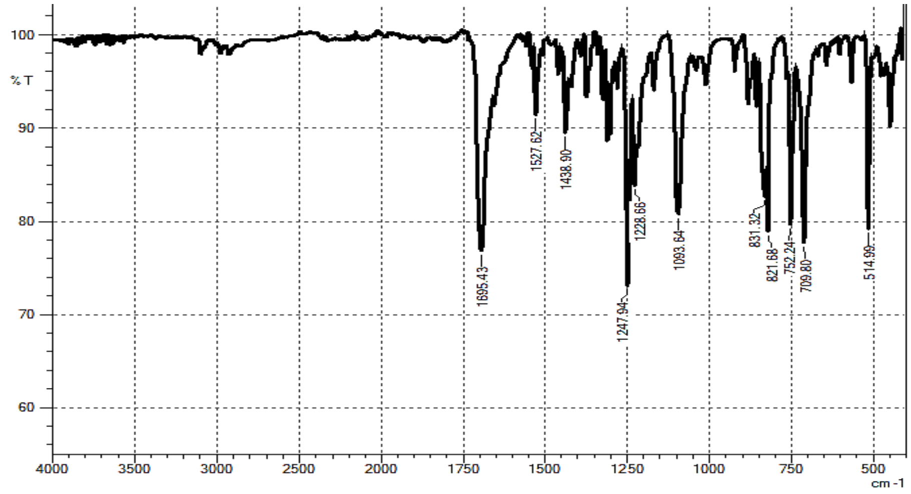

Figure S22. FTIR spectrum of **TBTE**.

1.1.5. Synthesis of ET5PtTBT

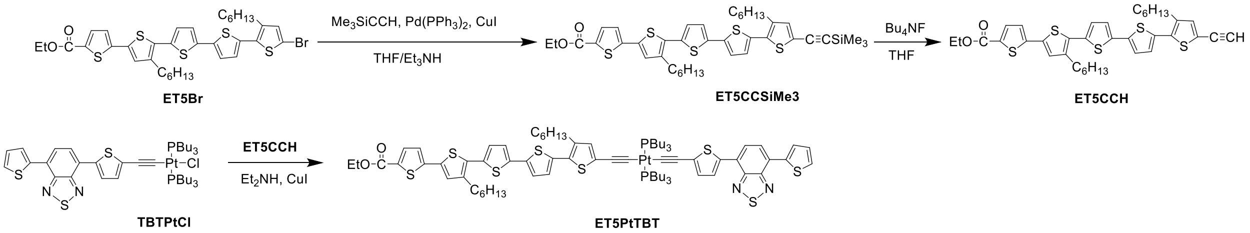

Scheme S5. Synthesis of **ET5PtTBT**

Synthesis of ET5CCSiMe$_3$

To a solution of **ET5Br** (100 mg, 0.136 mmol) in THF/Et$_3$NH (8/2 mL) were added trimethylsilyl acetylene (38 µL, 0.273 mmol), Pd(PPh$_3$)$_2$Cl$_2$ (6 mg, 6 mol%) and CuI (1.5 mg, 6 mol%) under nitrogen atmosphere. The reaction was stirred for 20 h at 60 ^{o}C. Then the solvents were evaporated, and the residue was chromatographed by flash column (silica) using hexane/DCM (80/20). Yield: 0.095 g, 95%. ^{1}H NMR (500 MHz, (CDCl$_3$): δ 7.71 (d, 1H), 7.14-7.17 (b, 4H), 7.10 (s, 1H), 7.08 (d, 1H), 7.05 (d, 1H), 4.38 (q, 2H), 2.79 (t, 2H), 2.75 (t, 2H), 1.62-1.73 (b, 4H), 1.31-1.45 (b, 15H), 0.90-0.94 (b, 6H), 0.28 (s, 9H). ^{13}C NMR (125 MHz, CDCl$_3$): δ 162.07, 143.70, 140.80, 139.49, 137.05, 137.01, 135.67, 134.67, 134.61, 134.13, 134.08, 132.15, 131.74, 131.16, 128.09, 126.94, 126.80, 124.15, 124.09, 123.71, 120.99, 99.92, 97.47, 61.24, 31.65, 30.42, 30.37, 29.48, 29.26, 29.21, 29.13, 22.60, 14.37, 14.10, 0.12. M/z: Calc. for $C_{40}H_{48}O_2S_5Si$ (M/z): 748.2022, Found: 748.2011.

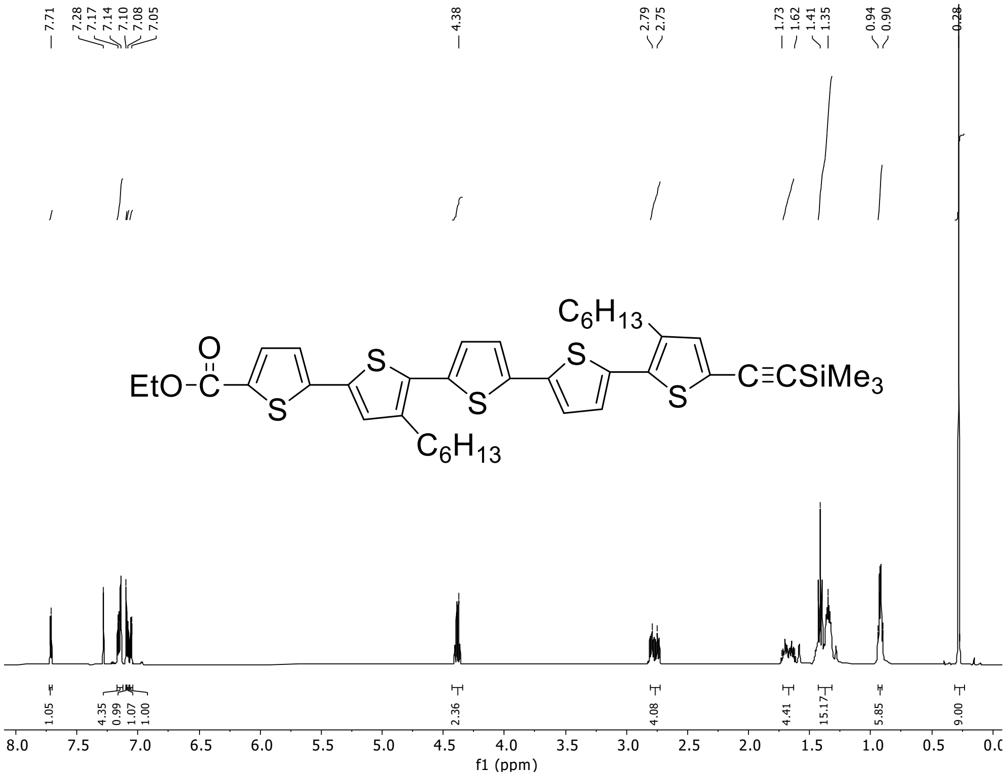

Figure S23. ^{1}H of **ET5CCSiMe₃**

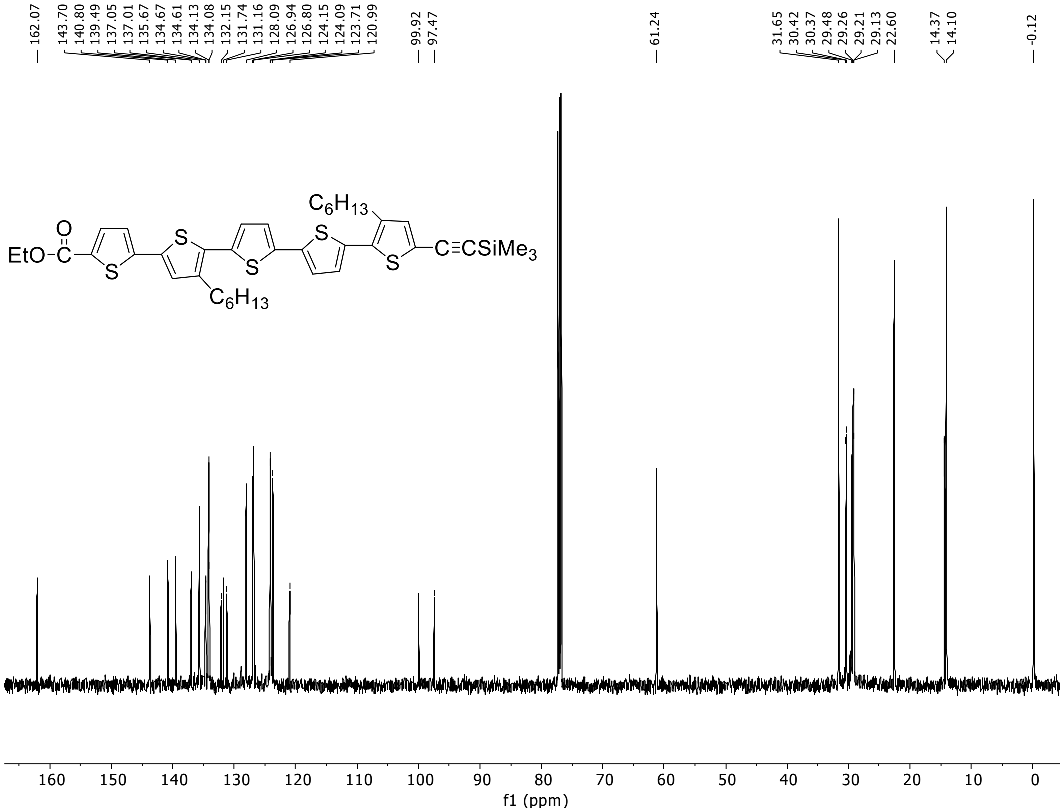

Figure S24. ^{13}C NMR of **ET5CCSiMe₃** in $CDCl_3$.

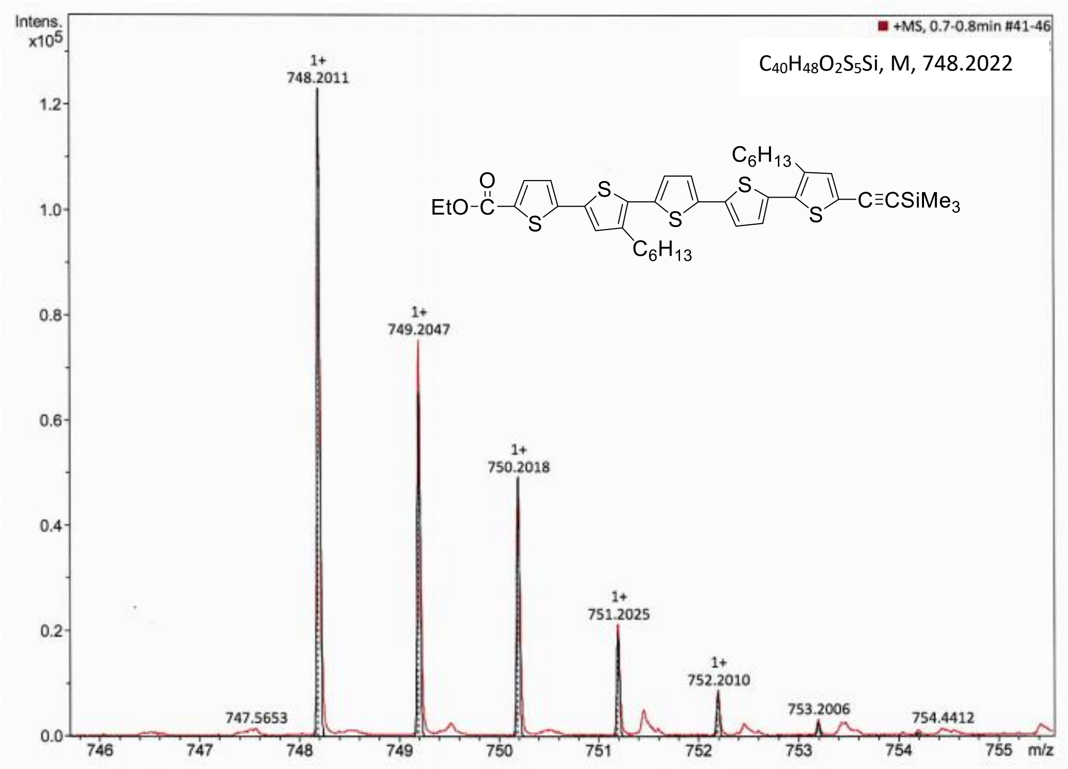

Figure S25. HRMS of **ET5CCSiMe₃** in $CDCl_3$.

Synthesis of ET5CCH

To a solution of **ET5CCSiMe₃** (80 mg, 0.107 mmol) in THF (5 mL) were added 1 M tetrabutyl ammonium fluoride (200 µL, 0.2 mmol) under nitrogen atmosphere. The reaction was stirred at 0 °C for 30 min. Then 2/3 drops of water was added and solvents were removed under reduced pressure, and the residue was chromatographed by flash column (silica) using hexane/DCM (60/40). Yield: 0.064 g, 89%. ^{1}H NMR (500 MHz, ($CDCl_3$): δ 7.71 (d, 1H), 7.13-7.16 (b, 5H), 7.08 (d, 1H), 7.06 (d, 1H), 4.39 (q, 2H), 4.31 (s, 1H), 2.79 (t, 2H), 2.75 (t, 2H), 1.63-1.73) (b, 4H), 1.39-1.47 (b, 7H), 1.32-1.37 (b, 8H), 0.91-0.94 (b, 6H). ^{13}C NMR (125 MHz, $CDCl_3$): δ 162.08, 143.70, 140.80, 139.49, 137.18, 136.98, 136.05, 134.73, 134.36, 134.13, 134.09, 132.42, 131.74, 131.15, 128.09, 127.09, 126.78, 124.18, 124.07, 123.71, 119.82, 82.11, 76.94, 61.24, 31.65, 30.42, 30.39, 29.50, 29.23, 29.14, 22.62, 14.38, 14.11. M/z: Calc. for $C_{37}H_{40}O_2S_5$ (M/z): 676.1631, Found: 676.1629.

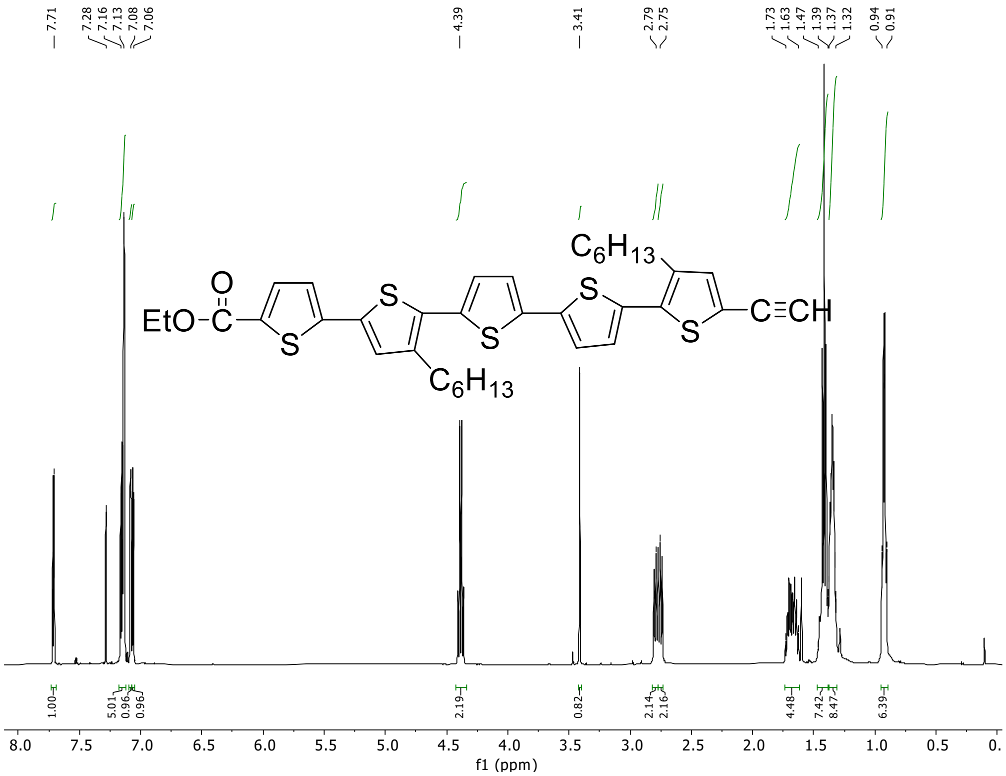

Figure S26. ^{1}H NMR of **ET5CCH** in $CDCl_3$.

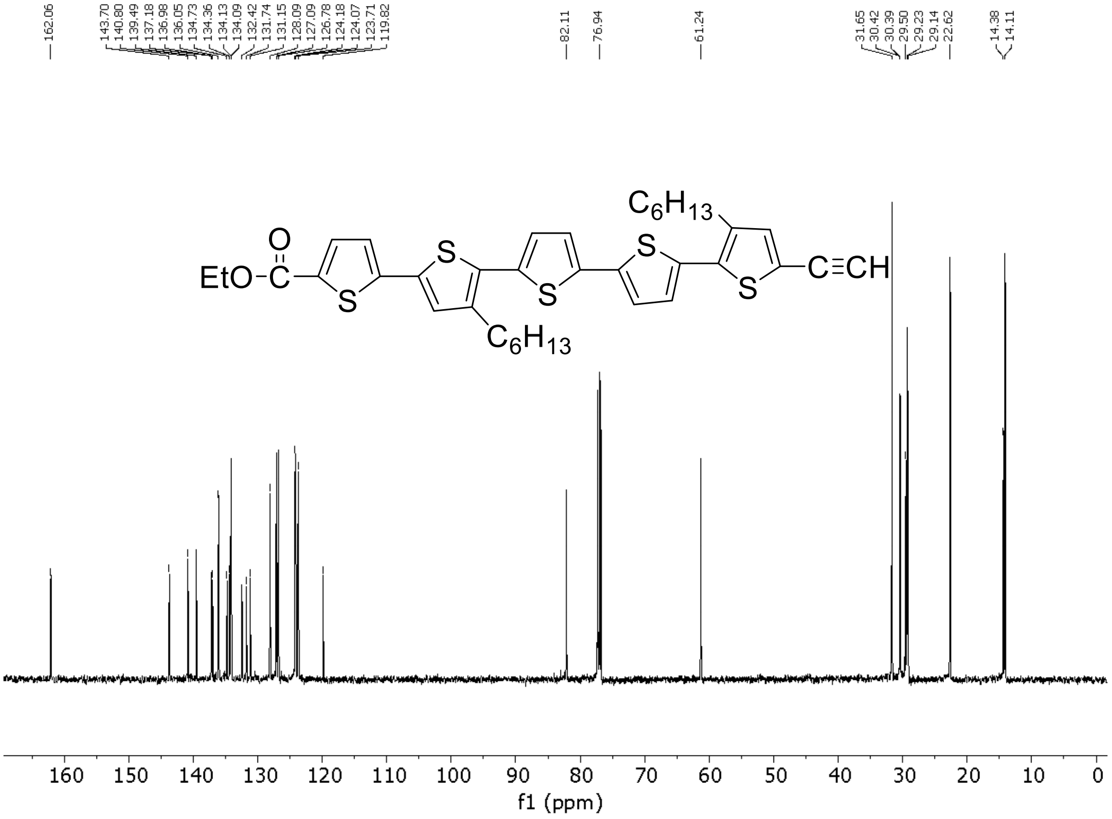

Figure S27. ^{13}C NMR of **ET5CCH** in $CDCl_3$.

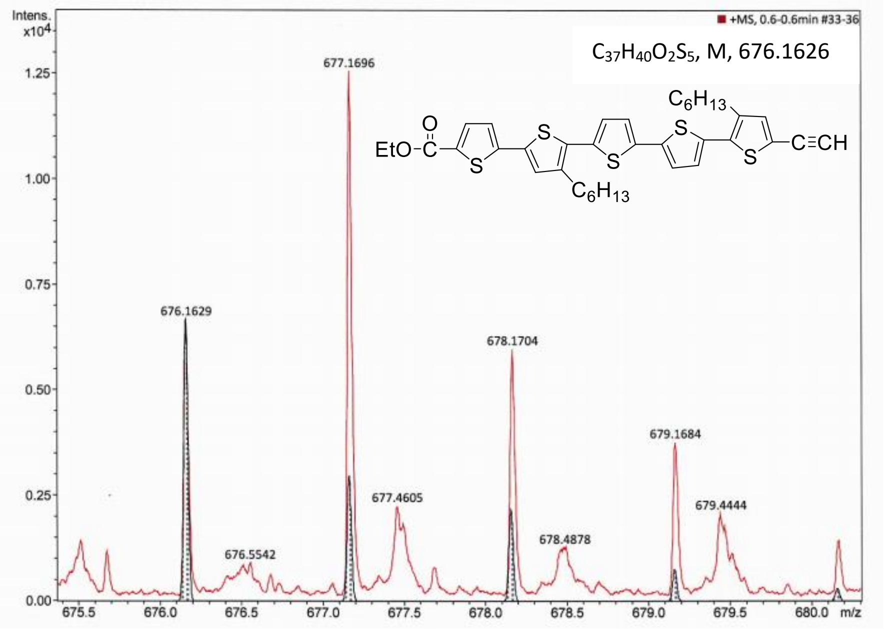

Figure S28. HRMS of **ET5CCH.**

Synthesis of ET5PtTBT

A mixture of **TBTPtCl** (54 mg, 0.056 mmol), ET5CCH (32 mg, 0.047 mmol) and CuI (1 mg) in Et_2NH was refluxed under nitrogen atmosphere for 18 h. The residue, after removal of the solvent, was purified using flash column with DCM/hexane as eluant. Yield: 35 mg, 46%. ^1H NMR (500 MHz, ($CDCl_3$): δ 8.12 (d, 1H), 8.02 (d, 1H), 7.86 (d, 1H), 7.77 (d, 1H), 7.71 (d, 1H), 7.46 (4, 1H), 7.23 (t, 1H), 7.14 (m, 4H), 7.07 (d, 1H), 7.99 (d, 1H), 6.94 (d, 1H), 6.71 (s, 1H), 4.37 (q, 2H), 2.79 (t, 2H), 2.73 (t, 2H), 2.15 (b, 12H), 1.65 (b, 16 H), 1.64 (b, 12 H), 1.53 (b, 6H), 1.35 (b, 9H), 0.99 (t, 18H), 0.93 (b, 6H). ^{13}C NMR (125 MHz, $CDCl_3$): δ 162.12, 152.78, 152.64, 143.79, 140.68, 139.64, 139.39, 137.58, 136.22, 135.87, 135.60, 134.16, 133.94, 131.67, 131.57, 131.36, 130.56, 128.54, 128.12, 128.00, 127.90, 127.26, 127.18, 126.82, 126.50, 126.33, 126.05, 125.68, 125.10, 124.71, 124.09, 123.72, 123.69, 101.94, 101.42, 61.24, 31.72, 31.64, 30.44, 30.38, 29.48, 29.43, 29.21, 26.40, 24.43, 24.05, 22.61, 14.38, 14.13, 14.10, 13.90. ^{31}P NMR (202 MHz, $CDCl_3$): δ 3.46 ($J_{(Pt-P)}$ = 2323 Hz). M/2z : Calc. for $C_{77}H_{100}N_2O_2P_2PtS_8$ (M/2z): 798.7336, Found: 798.7330.

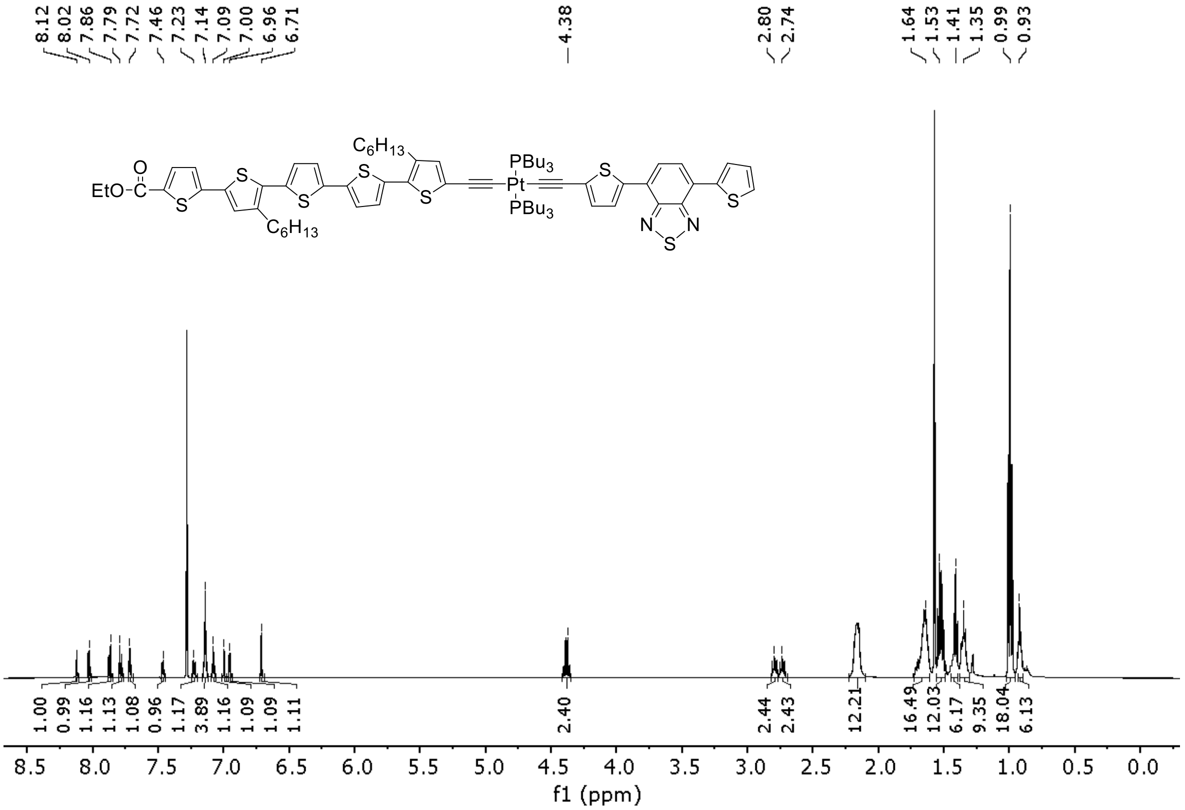

Figure S29a. ^{1}H NMR of **ET5PtTBT** in $CDCl_3$.

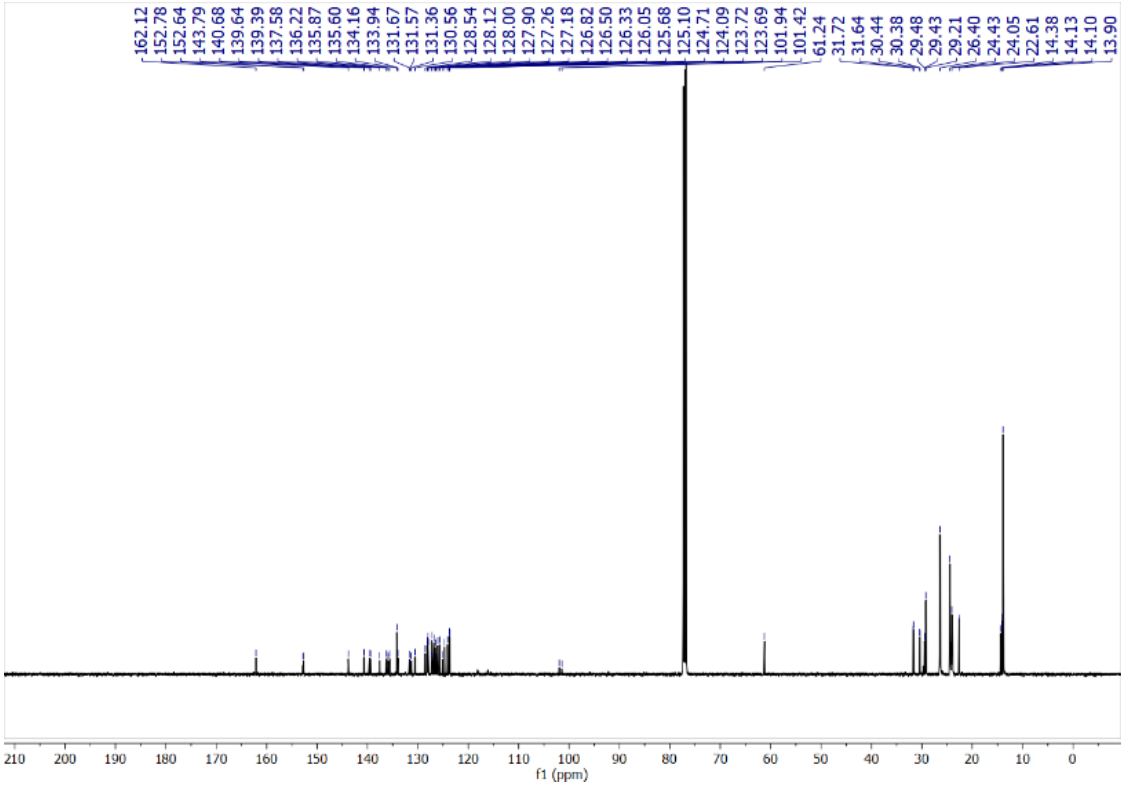

Figure S29b. ^{13}C NMR of **ET5PtTBT** in $CDCl_3$.

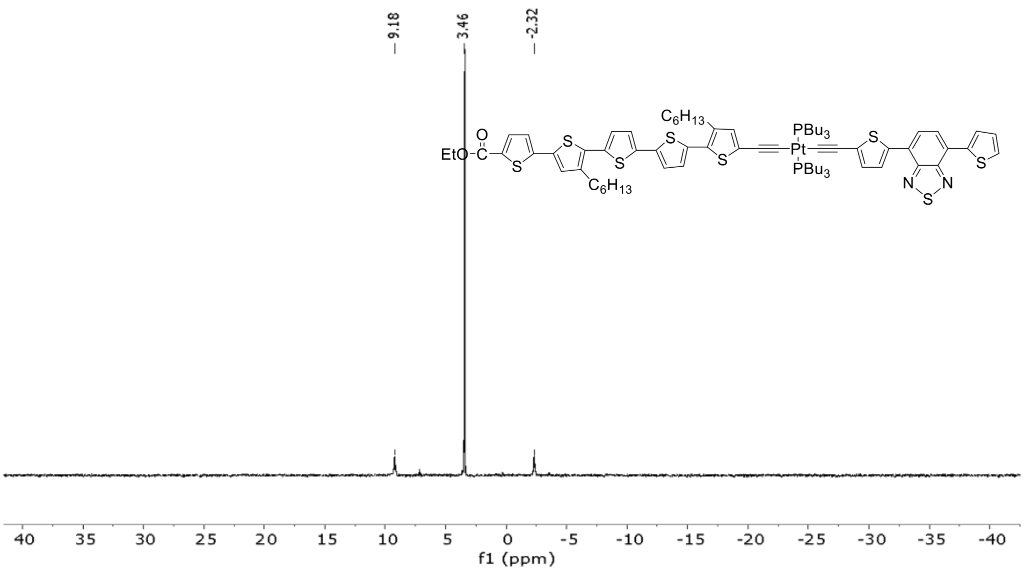

Figure S30. ^{31}P NMR of **ET5PtTBT** in $CDCl_3$.

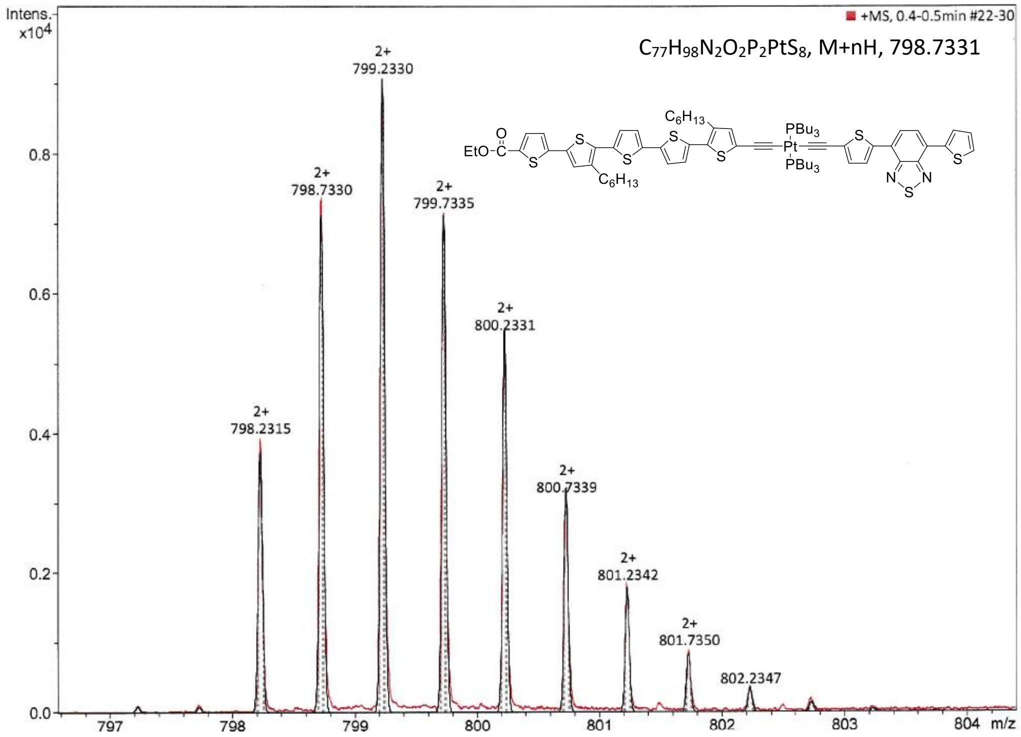

Figure S31. HRMS of **ET5PtTBT.**

Synthesis of T4PtTBTE

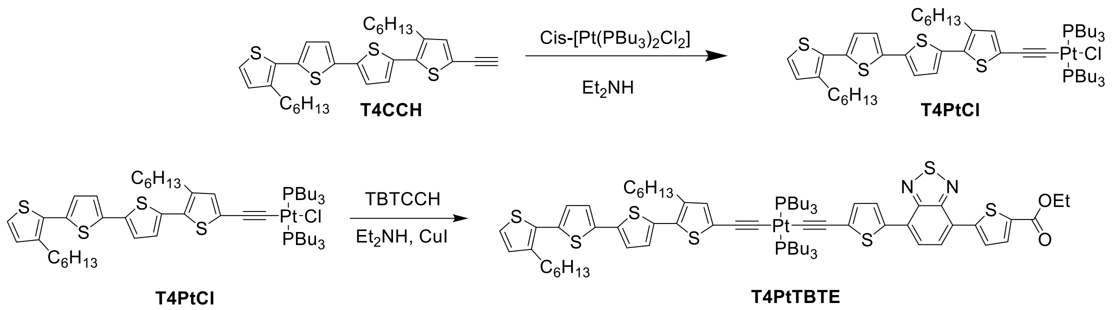

Scheme S6. Synthesis of T4PtTBTE.

Synthesis of T4PtCl

T4CCH (86 mg, 0.164 mmol) and cis-[$PtCl_2(PBu_3)_2$] (0.344 mg, 0.512 g) were added to degassed Et_2NH in a Schlenk flask, and the solution was stirred for 3 h at 60°C. Then the solvent was removed under reduced pressure and the crude product was purified by flash column (silica) chromatography using hexane/ DCM (70/30). Yield: 140 mg, 81%. ^{1}H NMR (500 MHz, ($CDCl_3$): δ 7.19 (d, 1H), 7.13 (d, 1H), 7.12 (d, 1H), 7.03 (d, 1H), 6.98 (d, 1H), 6.96 (d, 1H), 6.69 (s, 1H), 2.81 (t, 2H), 2.73 (t, 2H), 2.04 (b, 12H), 1.53-1.57 (b, 14H), 1.46 (m, 12H), 1.40 (b, 5H), 1.34 (b, 9H), 0.97 (t, 18H), 0.91 (b, 6H). M/z : Calc. for $C_{77}H_{100}N_2O_2P_2PtS_8$ (M/z): 1155.4502, Found: 1155.4505.

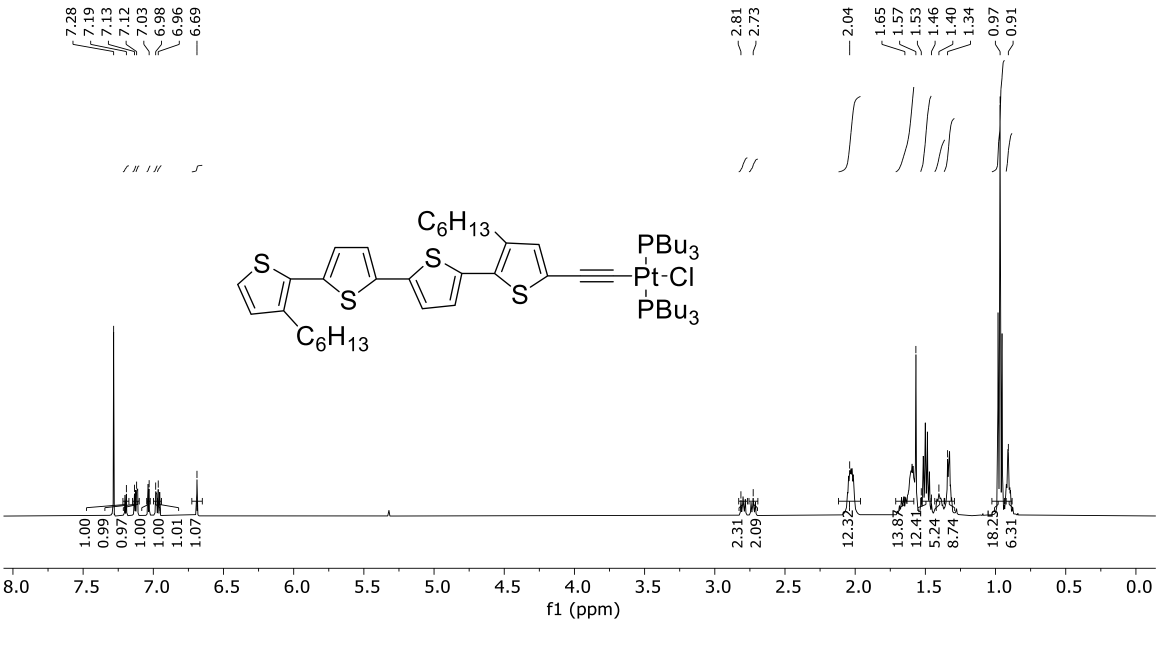

Figure S32. ^{1}H NMR of **T4PtCl** in $CDCl_3$.

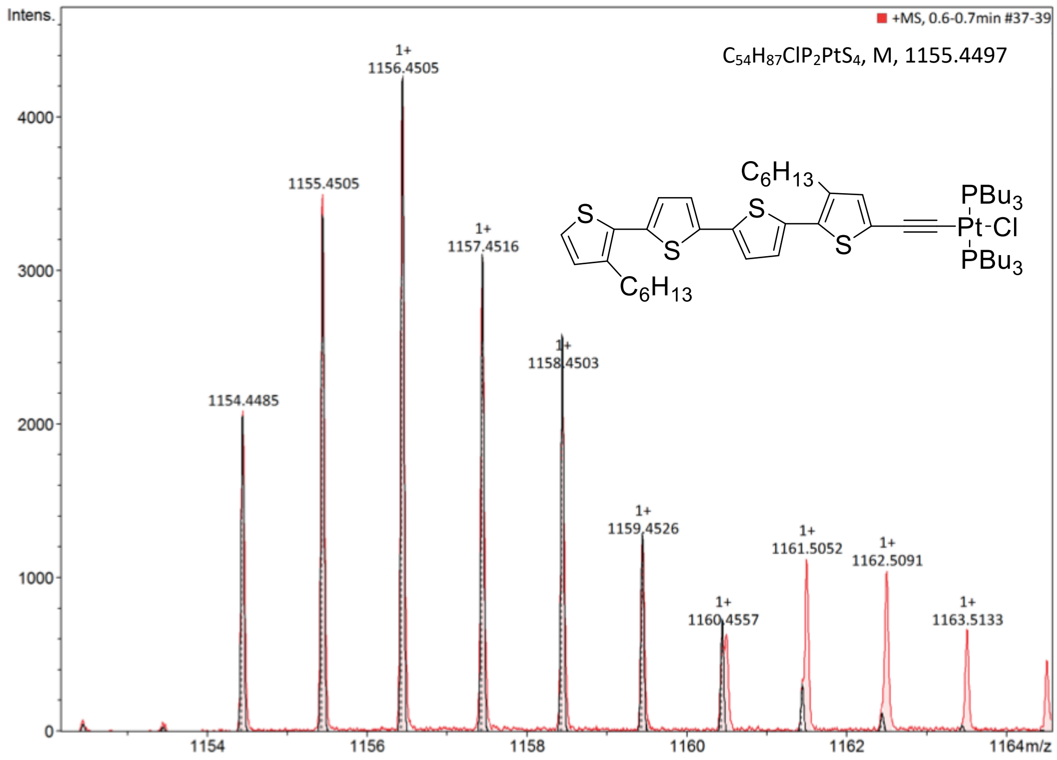

Figure S33. HRMS of **T4PtCl.**

Synthesis of T4PtTBTE

A mixture of **T4PtCl** (56 mg, 0.048 mmol), ETBTCCH (20 mg, 0.050 mmol) and CuI (1 mg, 0.005 mmol) in Et_2NH was stirred for 18 h, at 50° C, under nitrogen atmosphere. After removal of the solvent under reduced pressure, the crude product was purified by flash column (silica) chromatography using hexane/ DCM (60/30). Yield: 50 mg, 68% . ^{1}H NMR (500 MHz, ($CDCl_3$): δ 8.06 (d, 1H), 8.04 (d, 1H), 7.94 (d, 1H), 7.88 (d, 1H), 7.79 (d, 1H), 7.20 (d, 1H), 7.11 (b, 2H), 7.03 (m, 1H), 6.99 (b, 1H), 6.96 (d, 1H), 6.71 (s, 1H), 4.35 (q, 2H), 2.79 (t, 2H), 2.73 (t, 2H), 2.16 (b, 12H), 1.64 (b, 16 H), 1.53 (b, 12 H), 1.45 (b, 6H), 1.33 (b, 9H), 1.01 (t, 18H), 0.91 (b, 6H). ^{31}P NMR (202 MHz, $CDCl_3$): δ 3.44 ($J_{(Pt-P)}$ = 2320 Hz). M/2z : Calc. for $C_{73}H_{98}N_2O_2P_2PtS_7$ (M/2z): 757.7398, Found: 757.7398.

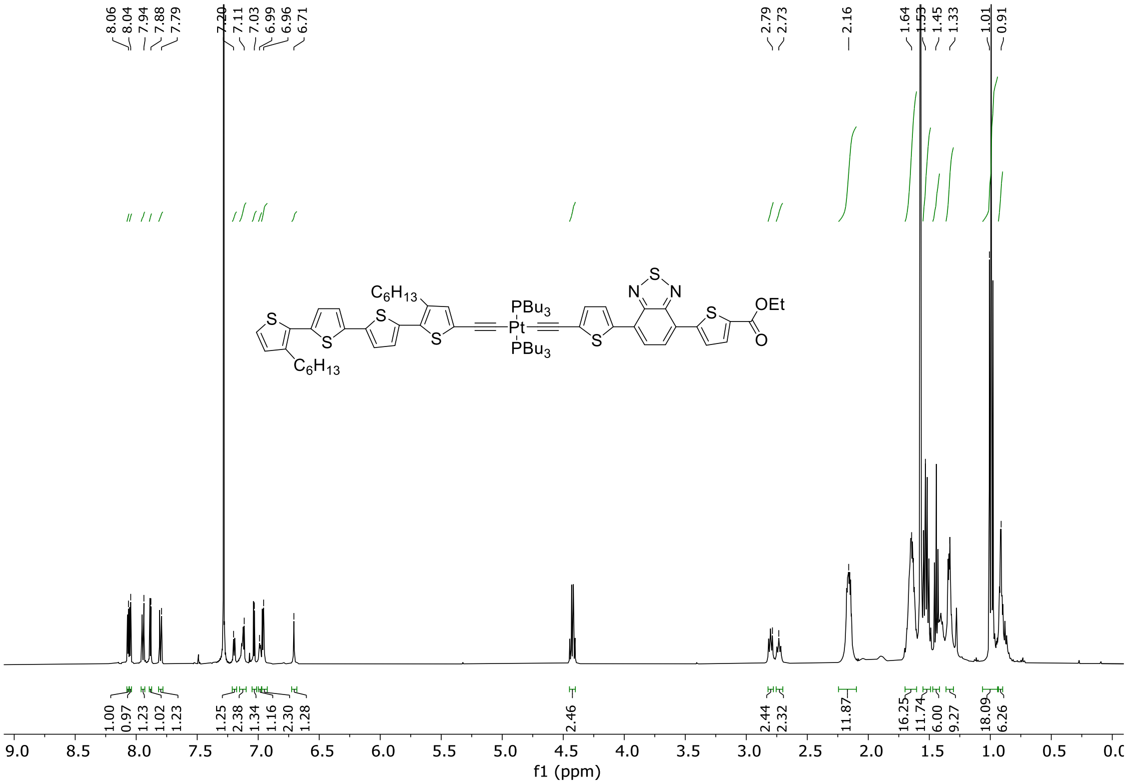

Figure S34. ^{1}H NMR of **T4PtTBTE** in $CDCl_3$.

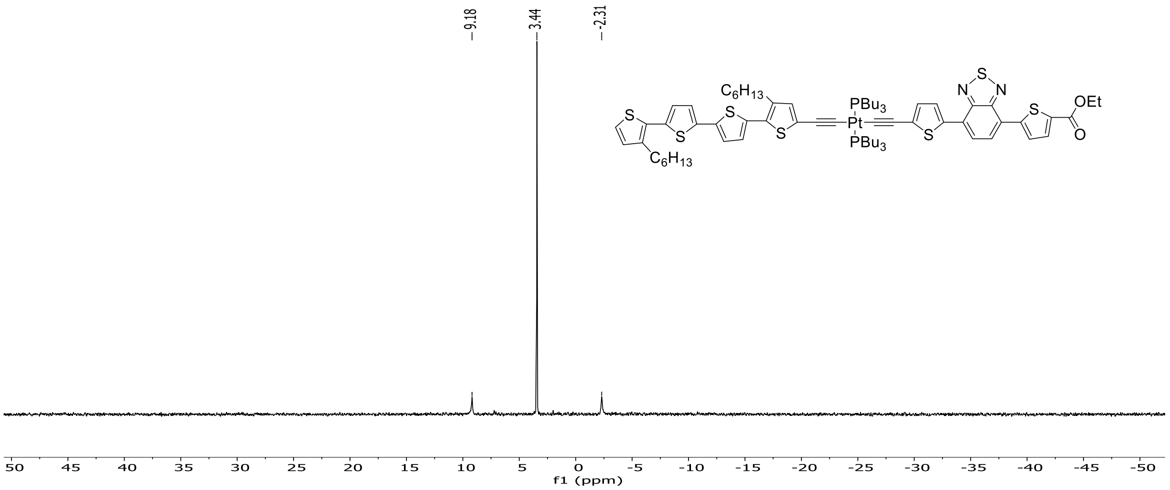

Figure S35. ^{31}P NMR of **T4PtTBTE** in $CDCl_3$.

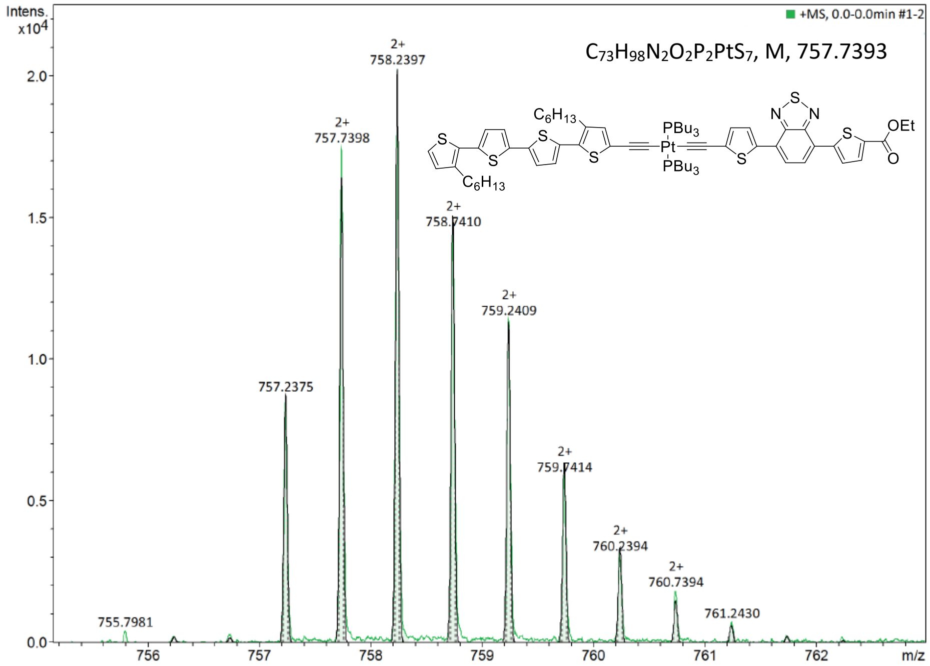

Figure S36. HRMS of **T4PtTBTE**

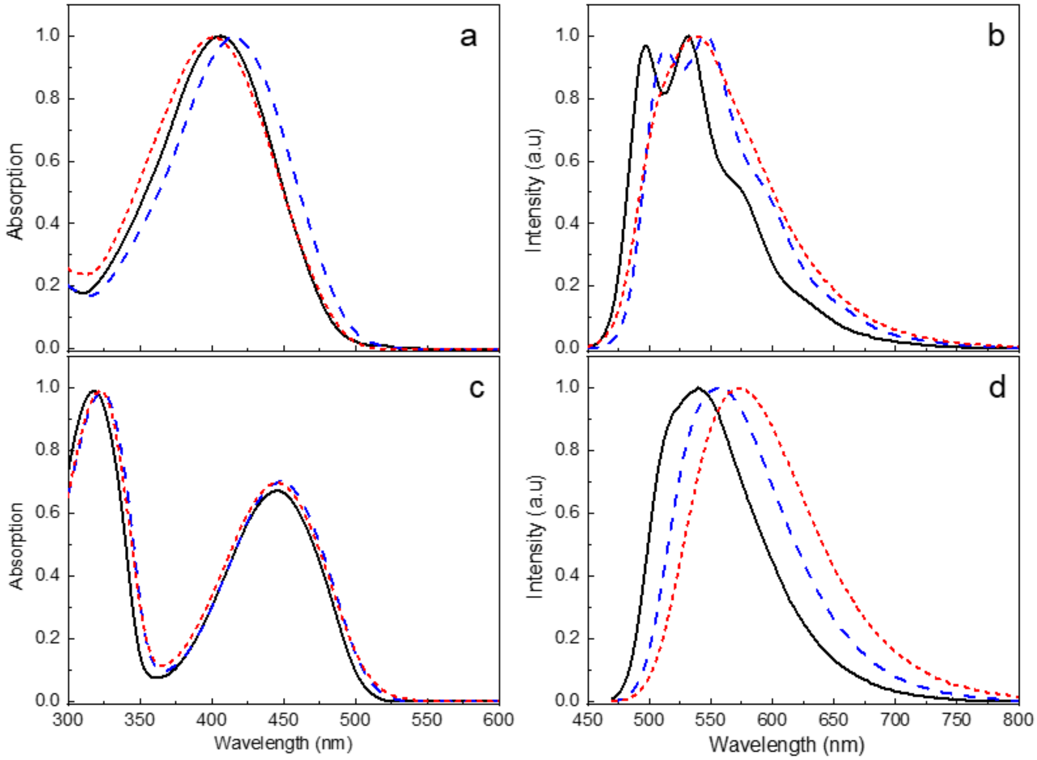

Figure S37. Normalized absorption and fluorescence spectra in three solvents. (a) **ET5** absorption; (b) **ET5** fluorescence. (c) **TBTE** absorption; (d) **TBTE** fluorescence. Solvents: hexane (black, solid line), Toluene (blue, dash line), and DCM (red, short dash line). The fluorescence emission measurements were obtained with excitation wavelength of 410 nm.

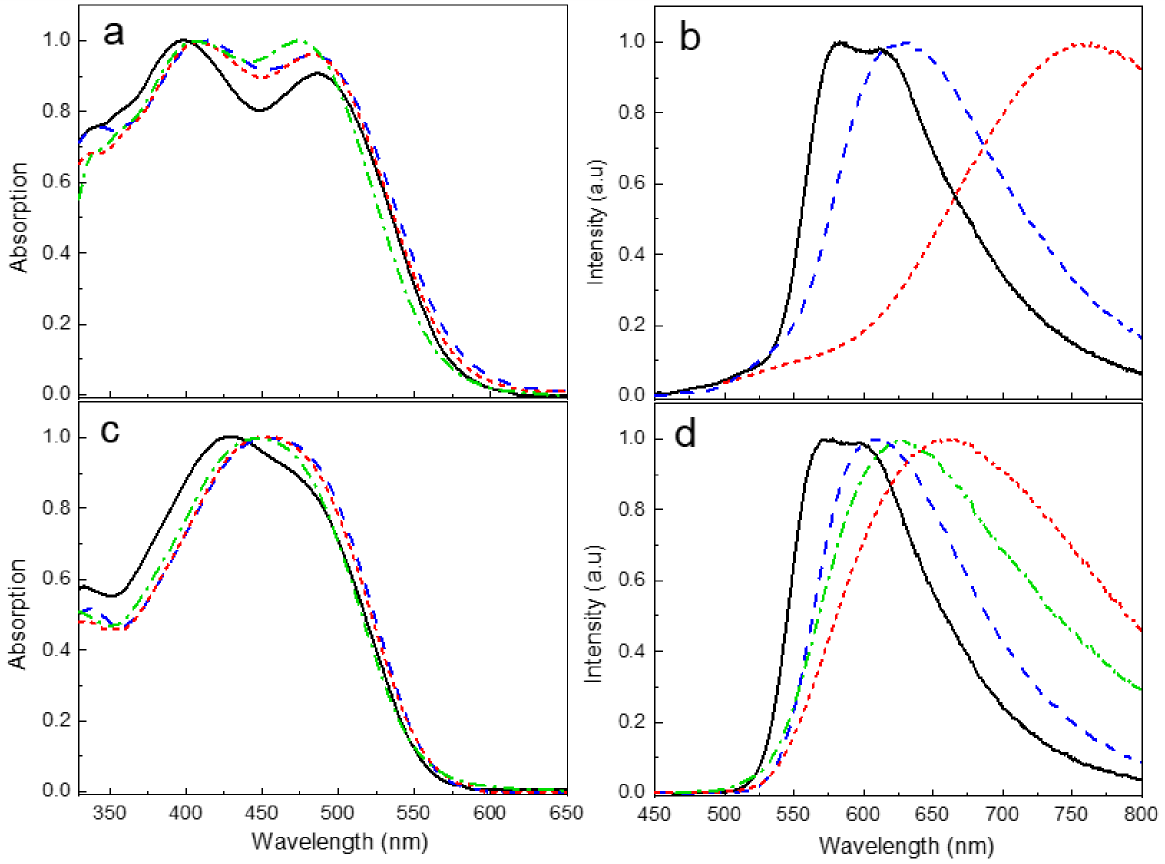

Figure S38. Absorption and fluorescence spectra in different solvents. (a) **T4TBTE** absorption. (b) **T4TBTE** fluorescence. (c) **ET5TBT** absorption. (d) **ET5TBT** fluorescence. Solvents: hexane (black, solid line), toluene (blue, dash line) dichloromethane (red, short dash line), acetone (green, dash dot line). The fluorescence emission measurements were obtained with excitation wavelengths of 410 nm and 430 nm for **T4TBTE** and **ET5TBT**, respectively.

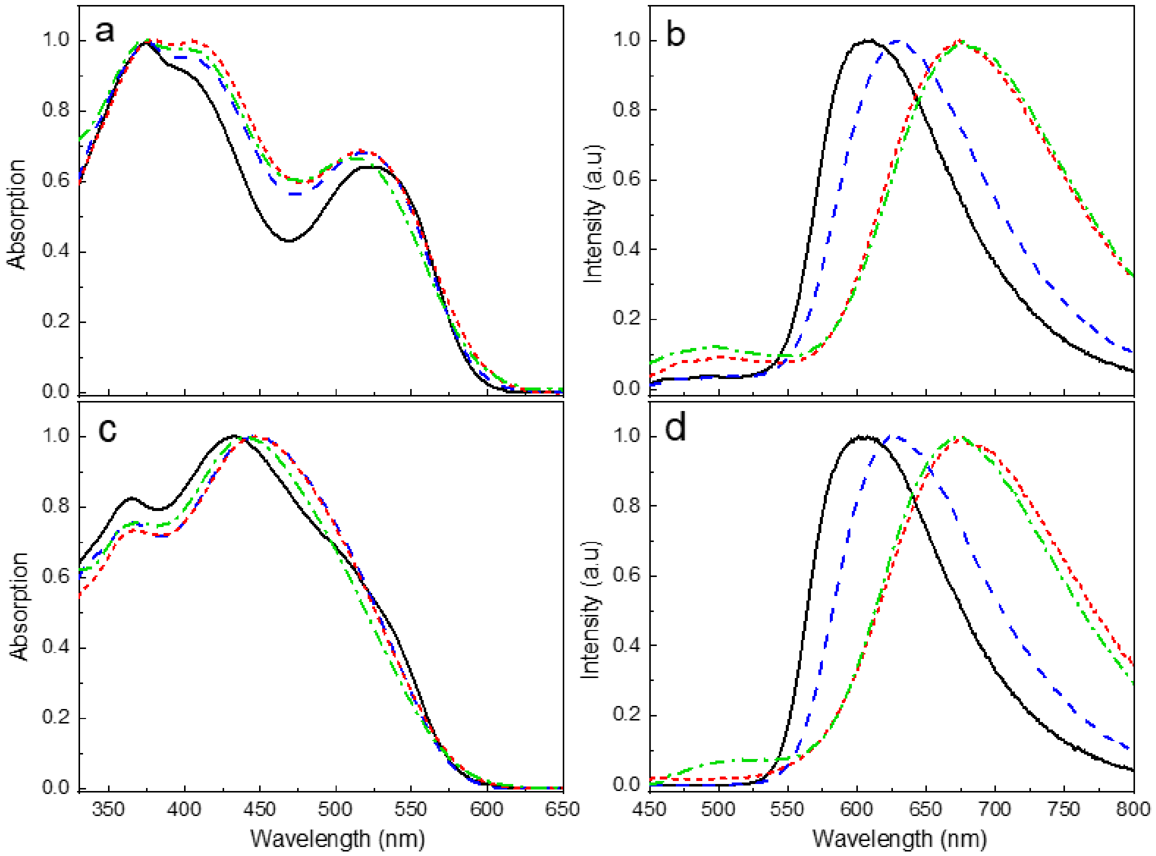

Figure S39. Absorption (a and c) and emission (b and d) of **T4PtTBTE** and **ET5PtTBT**, respectively, in hexane (black, solid line), toluene (blue, dash line) DCM (red, short dash line) and acetone (green, dash dot line). The fluorescence emission measurements were obtained with excitation wavelength of T4PtTBTE (400 nm), and ET5PtTBT (446 nm).

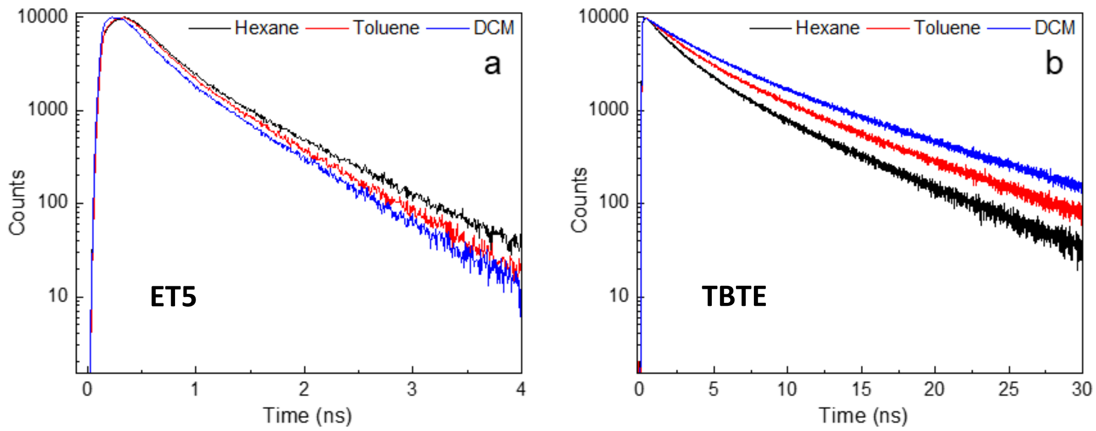

Figure S40. Fluorescence lifetime decays of **ET5** (a) and **TBTE** (b).

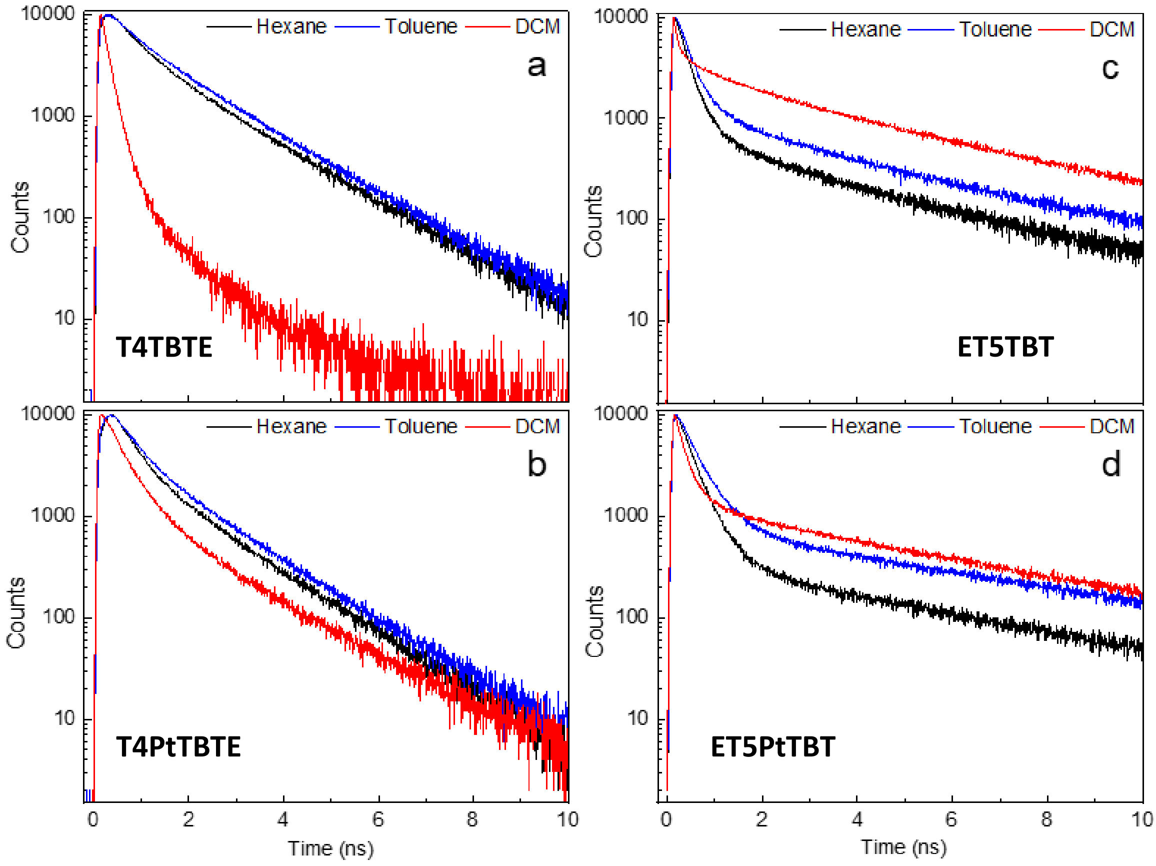

Figure S41. Fluorescence lifetime decays of **T4TBTE** (a), **ET5TBT** (b), **T4PtTBTE** (c), and **ET5PtTBT** (d).

Table S1. Fluorescence Decay Lifetimes [a]

Complexes	Hexane, τ (ns)			Toluene, τ (ns)			DCM, τ (ns)		
	τ_1	τ_2	$<\tau>$ [b]	τ_1	τ_2	$<\tau>$ [b]	τ_1	τ_2	$<\tau>$ [b]
**ET5**	0.25 (0.81)	0.76 (0.19)	0.35	0.3 (0.76)	0.69 (0.24)	0.39	0.24 (0.66)	0.65 (0.34)	0.38
**ETBT**	2.05 (0.66)	6.07 (0.34)	3.42	2.5 (0.6)	7.17 (0.4)	4.37	2.97 (0.54)	8.18 (0.46)	5.37
**T4TBTE**	0.56 (0.63)	1.57 (0.37)	0.93	0.62 (0.45)	1.61 (0.55)	1.16	0.16 (0.97)	0.77 (0.03)	0.18
**ET5TBT**	0.42 (0.84)	1.44 (0.16)	0.58	0.5 (0.49)	1.49 (0.51)	1.0	0.41 (0.88)	1.61 (0.12)	0.55
**T4PtTBTE**	0.25 (0.97)	3.64 (0.03)	0.35	0.28 (0.96)	4.0 (0.04)	0.43	0.17 (0.47)	3.93 (0.53)	2.16
**ET5PtTBT**	0.32 (0.97)	4.62 (0.03)	0.45	0.39 (0.66)	5.24 (0.34)	2.04	0.20 (0.91)	3.96 (0.09)	0.54

[a] Lifetimes in ns, relative amplitudes (α_i) in parenthesis. Excitation wavelengths: 450 nm for **ETBT** and 410 nm for all the other complexes. Detection wavelengths: i) **ET5**: 495 nm/ hexane, 510 nm/ toluene, and 515 nm/ DCM; ii) **ETBT**: 525 nm/ hexane, 545 nm/ toluene, and 562 nm/ DCM; iii) **T4TBTE**: 575 nm/ hexane, 605 nm/ toluene, and 680 nm/ DCM; iii) **ET5TBT**: 563 nm/ hexane, 590 nm/ toluene, and 622 nm/ DCM; iii) **T4PtTBTE**: 590 nm/ hexane, 610 nm/ toluene, and 660 nm/ DCM; iii) **ET5PtTBT**: 585 nm/ hexane, 608 nm/ toluene, and 650 nm/ DCM.
[b] $<\tau> = \Sigma\alpha_i\tau_i$.

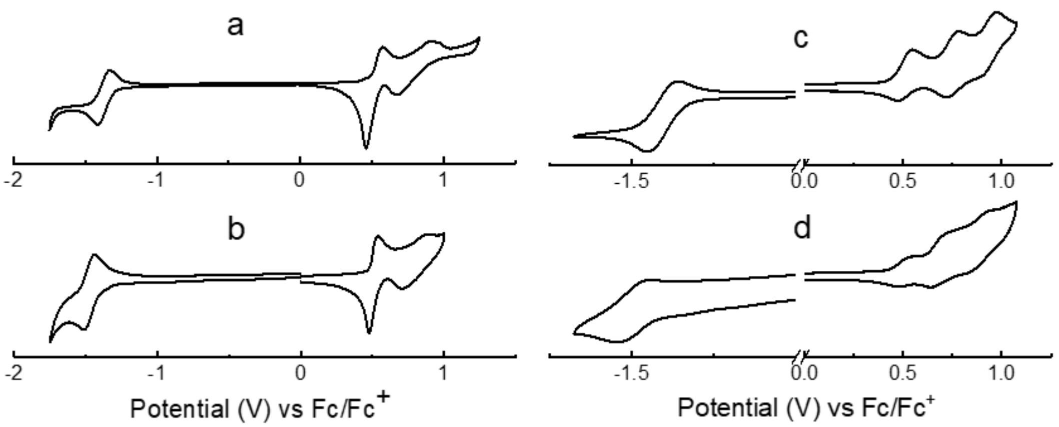

Figure S42. Cyclic voltammograms of **T4TBTE** (a), **ET5TBT** (b), **T4PtTBTE** (c), and **ET5TBT** measured in nitrogen saturated anhydrous dichloromethane with 0.1 M tetrabutyl ammonium hexafluoro phosphate as a supporting electrolyte in a three-electrode set up with glassy carbon (WE), silver/silver chloride (**T4TBTE** and **ET5TBT**) and silver/silver nitrate (**T4PtTBTE** and **ET5PtTBT**) (RE), and platinum wire (CE) and a scan rate of 100 mVs^{-1}. The redox values are referenced vs Fc/Fc^+ potential.

Table S2. Electrochemical data[a] and calculated HOMO/LUMO energies[b].

Compound	$E_{1/2}$ Rd	$E_{1/2}$ Ox	HOMO (eV)	LUMO (eV)
ET5TBT	-1.48	0.51	-5.31	-3.32
T4TBTE	-1.37	0.52	-5.32	-3.43
ET5PtTBT	-1.5	0.50	-5.3	-3.3
T4PtTBTE	-1.4	0.51	-5.31	-3.4

[a] Measured in nitrogen saturated dichloromethane with 0.1 M tetrabutyl ammonium hexafluoro phosphate as a supporting electrolyte in a three-electrode set up with glassy carbon (WE), silver/silver chloride (**T4TBTE** and **ET5TBT**) and silver/silver nitrate (**T4PtTBTE** and **ET5PtTBT**) (RE), and platinum wire (CE) and a scan rate of 100 mVs^{-1}. The redox values are referenced vs Fc/Fc^+ potential. [b] HOMO and LUMO energies are calculated from the $E_{1/2}$ Ox and $E_{1/2}$ Rd vs Fc/Fc^+ using the formula E_{HOMO} (eV) = - ($E_{1/2}$ Ox + 4.8) and E_{LUMO} (eV) = - ($E_{1/2}$ Rd + 4.8).

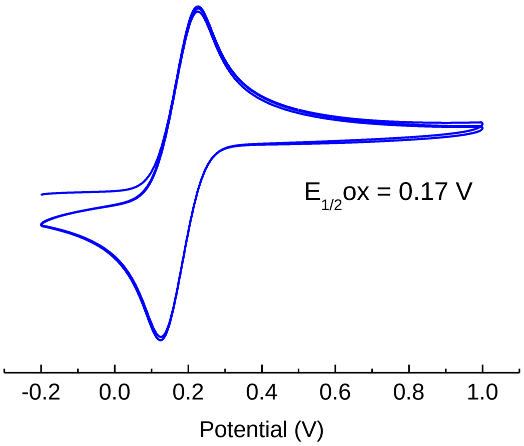

Figure S43. Cyclic voltammogram of ferrocene measured in nitrogen saturated anhydrous dichloromethane with 0.1 M tetrabutylammonium hexafluorophosphate as a supporting electrolyte in a three-electrode set up with glassy carbon (WE), silver/silver chloride (RE) and platinum wire (CE), and a scan rate of 100 mVs^{-1}.

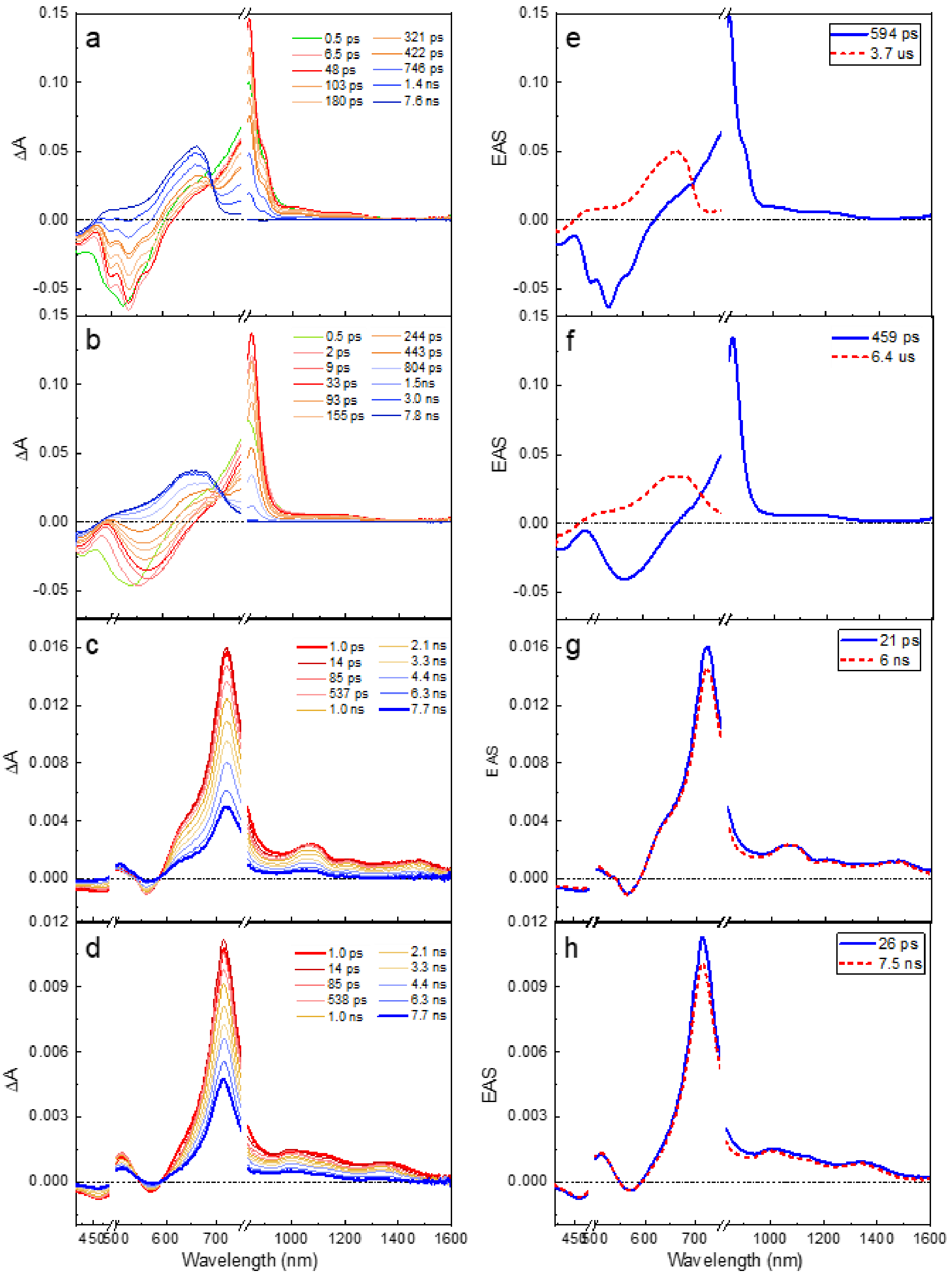

Figure S44. fsTA and evolution associated spectra (EAS), respectively, of **ET5** in hexane (a and e) and in DCM (b and f) at λ_{ex} = 420 nm and the same data for **TBTE** in hexane (c and g) and in DCM (d and h) at λ_{ex} = 490 nm.

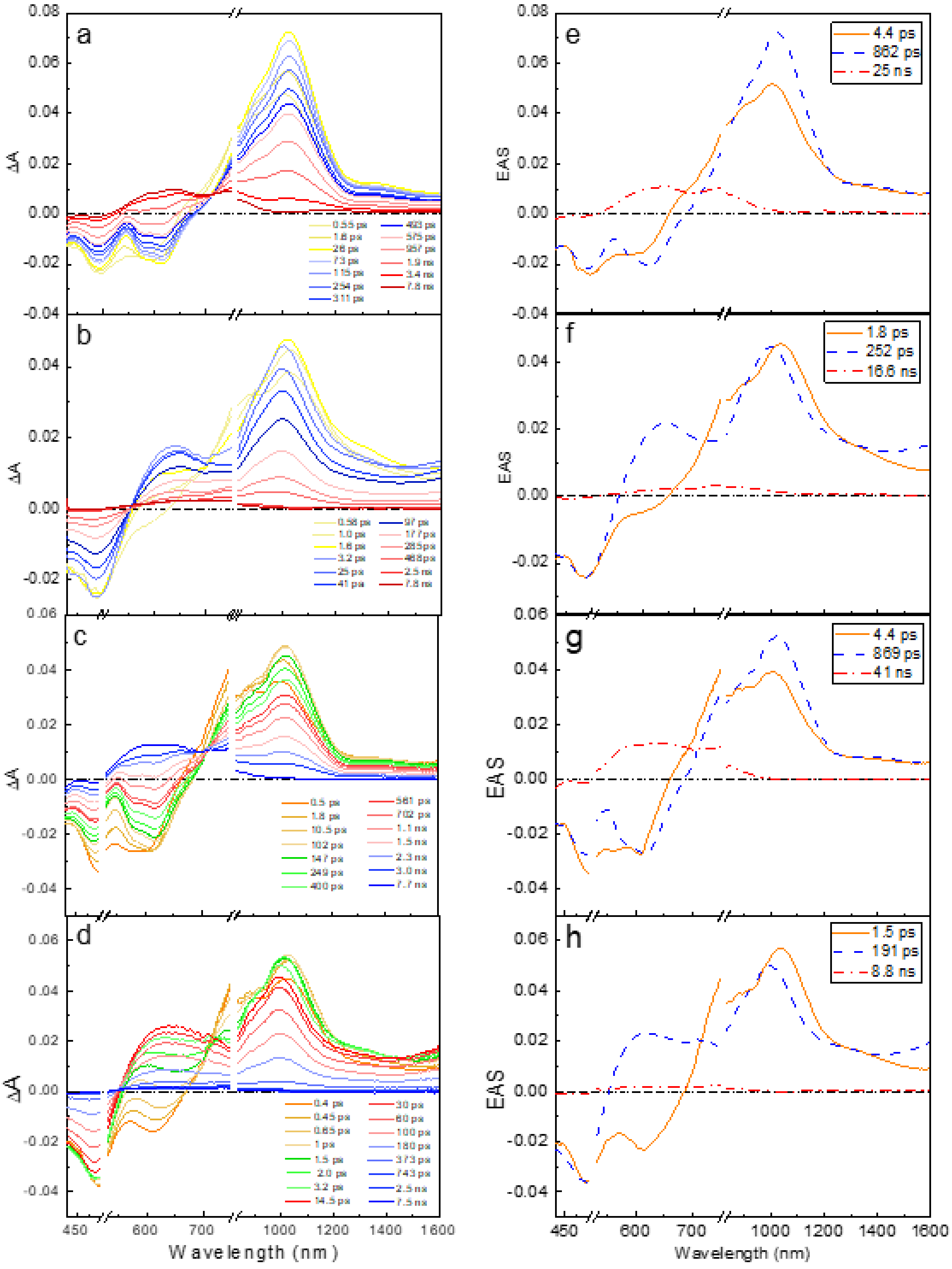

Figure S45. fsTA and evolution associated spectra (EAS), respectively, of **T4TBTE** in hexane (a and e) and DCM (b and f) at λ_{ex} = 420 and the same data at λ_{ex} = 520 in hexane (c and g) and DCM (d and h)

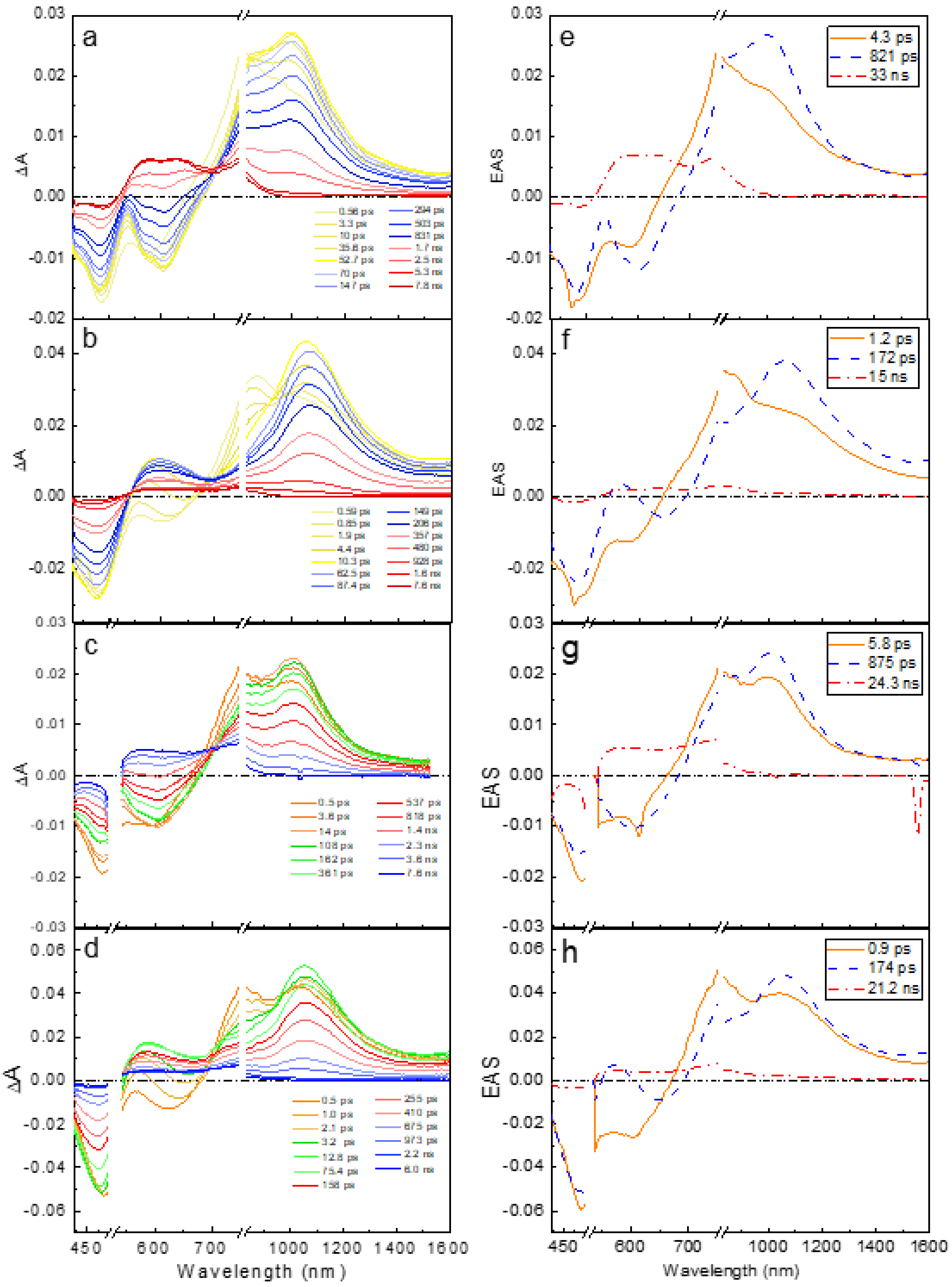

Figure S46. fsTA and evolution associated spectra (EAS), respectively, of **ET5TBT** in hexane (a and e) and DCM (b and f) at $\lambda_{ex} = 420$ and the same data at $\lambda_{ex} = 520$ in hexane (c and g) and DCM (d and h).

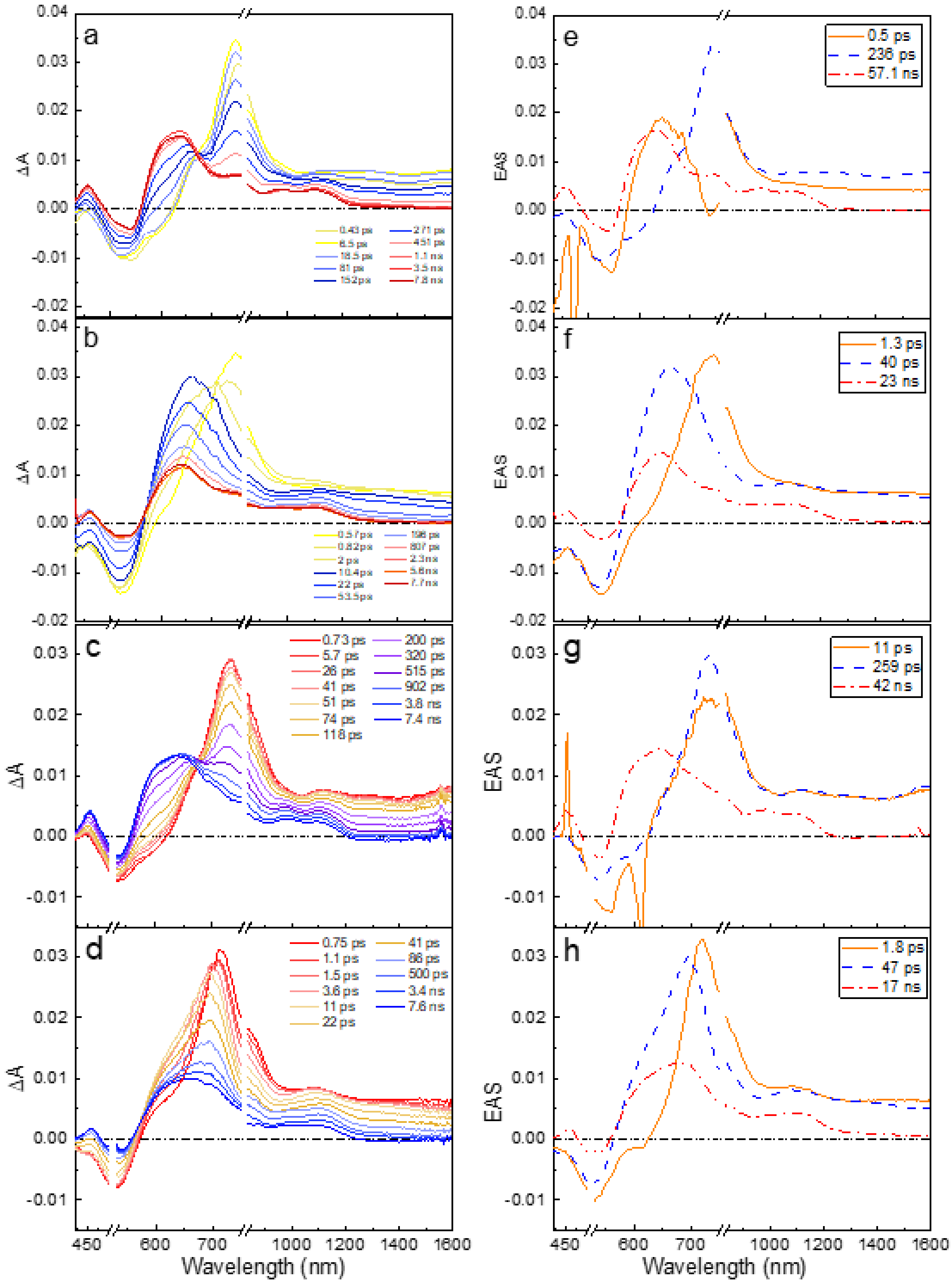

Figure S47. fsTA and evolution associated spectra (EAS), respectively, of **T4PtTBT** in hexane (a and e) and DCM (b and f) at $\lambda_{ex} = 420$ and the same data at $\lambda_{ex} = 520$ in hexane (c and g) and DCM (d and h)

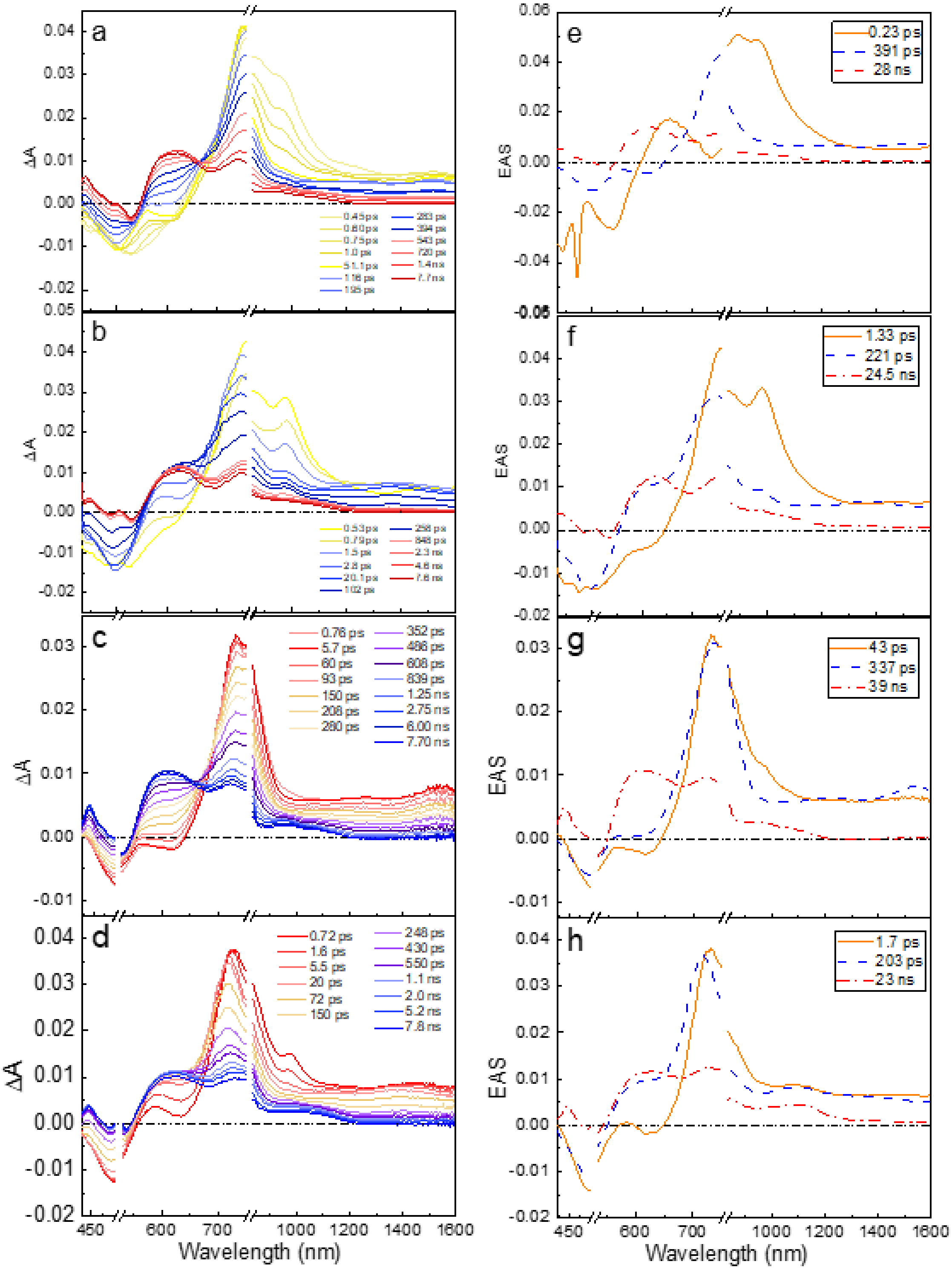

Figure S48. fsTA and evolution associated spectra (EAS), respectively, of **ET5PtTBT** in hexane (a and e) and DCM (b and f) at $\lambda_{ex} = 420$ and the same data at $\lambda_{ex} = 520$ in hexane (c and g) and DCM (d and h).

References for Supplementary Materials

1. Chen, Z.; Grumstrup, E. M.; Gilligan, A. T.; Papanikolas, J. M.; Schanze, K. S., Light-Harvesting Polymers: Ultrafast Energy Transfer in Polystyrene-Based Arrays of Π-Conjugated Chromophores. *J. Phys. Chem. B* **2014,** *118,* 372-378.

2. Jones, A. L.; Gish, M. K.; Zeman, C. J. I. V.; Papanikolas, J. M.; Schanze, K. S., Photoinduced Electron Transfer in Naphthalene Diimide End-Capped Thiophene Oligomers. *J. Phys. Chem. A* **2017,** *121*, 9579-9588.

3. Lu, C.; Fujitsuka, M.; Majima, T., Photoaccelerated Hole Transfer in Oligothiophene Assemblies. *The Journal of Physical Chemistry C* **2017,** *121*, 649-655.

4. Liu, Q.; Zhu, N.; Ho, C.-L.; Fu, Y.; Lau, W.-S.; Xie, Z.; Wang, L.; Wong, W.-Y., Synthesis, Characterization, Photophysical and Photovoltaic Properties of New Donor–Acceptor Platinum(Ii) Acetylide Complexes. *J. Organomet. Chem.* **2016,** *812*, 2-12.

5. Akkuratov, A. V.; Susarova, D. K.; Kozlov, O. V.; Chernyak, A. V.; Moskvin, Y. L.; Frolova, L. A.; Pshenichnikov, M. S.; Troshin, P. A., Design of (X-Dadad)N Type Copolymers for Efficient Bulk Heterojunction Organic Solar Cells. *Macromolecules* **2015,** *48*, 2013-2021.
